# Effectiveness of Genotype-Specific Tricyclic Antidepressant Dosing in Patients With Major Depressive Disorder: A Randomized Clinical Trial

**DOI:** 10.1001/jamanetworkopen.2023.12443

**Published:** 2023-05-08

**Authors:** Cornelis F. Vos, Sophie E. ter Hark, Arnt F. A. Schellekens, Jan Spijker, Annemarie van der Meij, Anne J. Grotenhuis, Raluca Mihaescu, Wietske Kievit, Rogier Donders, Rob E. Aarnoutse, Marieke J. H. Coenen, Joost G. E. Janzing

**Affiliations:** 1 Department of Psychiatry, Radboud University Medical Center, Nijmegen, the Netherlands; 2 Donders Institute for Brain, Cognition and Behavior, Radboud University, Nijmegen, the Netherlands; 3 Nijmegen Institute for Scientist Practitioners in Addiction, Radboud University, Nijmegen, the Netherlands; 4 Depression Expertise Centre, Pro Persona, Nijmegen, the Netherlands; 5 Behavioural Science Institute, Radboud University, Nijmegen, the Netherlands; 6 Dutch Depression Society, Amersfoort, the Netherlands; 7 Department of Psychiatry, Catharina Hospital, Eindhoven, the Netherlands; 8 Department of Health Evidence, Radboud University Medical Center, Nijmegen, the Netherlands; 9 Department of Pharmacy, Radboud University Medical Center, Nijmegen, the Netherlands; 10 Department of Clinical Chemistry, Erasmus University Medical Centre, Rotterdam, the Netherlands

## Abstract

**Importance:**

Evidence of the clinical benefit of pharmacogenetics-informed treatment (PIT) with antidepressants is still limited. Especially for tricyclic antidepressants (TCAs), pharmacogenetics may be of interest because therapeutic plasma concentrations are well defined, identification of optimal dosing can be time consuming, and treatment is frequently accompanied by adverse effects.

**Objective:**

To determine whether PIT results in faster attainment of therapeutic TCA plasma concentrations compared with usual treatment in patients with unipolar major depressive disorder (MDD).

**Design, Setting, and Participants:**

This randomized clinical trial compared PIT with usual treatment among 111 patients at 4 centers in the Netherlands. Patients were treated with the TCAs nortriptyline, clomipramine, or imipramine, with clinical follow-up of 7 weeks. Patients were enrolled from June 1, 2018, to January 1, 2022. At inclusion, patients had unipolar nonpsychotic MDD (with a score of ≥19 on the 17-item Hamilton Rating Scale for Depression [HAMD-17]), were aged 18 to 65 years, and were eligible for TCA treatment. Main exclusion criteria were a bipolar or psychotic disorder, substance use disorder, pregnancy, interacting comedications, and concurrent use of psychotropic medications.

**Intervention:**

In the PIT group, the initial TCA dosage was based on *CYP2D6* and *CYP2C19* genotypes. The control group received usual treatment, which comprised the standard initial TCA dosage.

**Main Outcomes and Measures:**

The primary outcome was days until attainment of a therapeutic TCA plasma concentration. Secondary outcomes were severity of depressive symptoms (measured by HAMD-17 scores) and frequency and severity of adverse effects (measured by Frequency, Intensity, and Burden of Side Effects Rating scores).

**Results:**

Of 125 patients randomized, 111 (mean [SD] age, 41.7 [13.3] years; 69 [62.2%] female) were included in the analysis; of those, 56 were in the PIT group and 55 were in the control group. The PIT group reached therapeutic concentrations faster than the control group (mean [SD], 17.3 [11.2] vs 22.0 [10.2] days; Kaplan-Meier *χ^2^*_1_ = 4.30; *P* = .04). No significant difference in reduction of depressive symptoms was observed. Linear mixed-model analyses showed that the interaction between group and time differed for the frequency (*F*_6,125_ = 4.03; *P* = .001), severity (*F*_6,114_ = 3.10; *P* = .008), and burden (*F*_6,112_ = 2.56; *P* = .02) of adverse effects, suggesting that adverse effects decreased relatively more for those receiving PIT.

**Conclusions and Relevance:**

In this randomized clinical trial, PIT resulted in faster attainment of therapeutic TCA concentrations, with potentially fewer and less severe adverse effects. No effect on depressive symptoms was observed. These findings indicate that pharmacogenetics-informed dosing of TCAs can be safely applied and may be useful in personalizing treatment for patients with MDD.

**Trial Registration:**

ClinicalTrials.gov Identifier: NCT03548675

## Key Points

**Question:**

Does pharmacogenetics-informed (*CYP2D6* and *CYP2C19* genotypes) dosing of tricyclic antidepressants result in faster attainment of therapeutic plasma concentrations, higher effectiveness, and fewer and less severe adverse effects compared with usual treatment?

**Findings:**

In this randomized clinical trial of 111 patients with major depressive disorder, dosing based on preemptive pharmacogenetic testing led to faster attainment of therapeutic plasma concentrations and potentially fewer and less severe adverse effects. No effect on depressive symptoms was found.

**Meaning:**

These findings indicate that pharmacogenetics-informed dosing of tricyclic antidepressants can be safely applied and contributes to personalized antidepressant treatment.

## Teaser

This randomized clinical trial examines whether tricyclic antidepressant (TCA) dosing based on pharmacogenetics according to the Dutch Pharmacogenetics Working Group guidelines results in faster attainment of therapeutic TCA plasma concentrations compared with usual treatment.

## Introduction

Pharmacogenetics has the potential to personalize antidepressant treatment, yet implementation in psychiatry is still very limited.^1,2,3,4^ To date, there is a lack of robust randomized clinical trials (RCTs) comparing pharmacogenetics-informed treatment (PIT) vs standard treatment. As a result, the benefit of pharmacogenetics in clinical practice remains unknown.^4,5^ Given the delayed onset of treatment response, high occurrence of nonresponse, and common adverse effects of antidepressants in the treatment of unipolar major depressive disorder (MDD), personalization of antidepressant pharmacotherapy is highly needed.^6,7,8^

Antidepressants are metabolized by various isoforms of cytochrome P450 (CYP450) enzymes, notably the CYP450 2D6 (CYP2D6) and CYP450 2C19 (CYP2C19) isozymes.^9^ The activity of these enzymes widely differs between individuals and largely depends on genetic variation in the genes coding for these enzymes.^1,2^ It has been reported that CYP2D6 and CYP2C19 metabolizer phenotypes determine exposure to antidepressant plasma concentrations.^10^ Personalizing the dosage by taking CYP450 activity into account may result in earlier attainment of therapeutic plasma concentrations and consequently promote effectiveness and prevent adverse effects.^11,12^ A number of studies have found that pharmacogenetic testing is associated with higher effectiveness of antidepressants for the treatment of MDD.^5,13,14,15,16^ However, most of those studies^13,15,16^ did not provide information on the interaction between antidepressant and genotype because multiple genes are included in combinatorial pharmacogenetic tests. In addition, prescribers were free to adhere to the test results, leaving the clinical benefit of dosing based on pharmacogenetics for a preselected antidepressant unclear.

Currently, several guidelines are available for optimizing pharmacotherapy by pharmacogenetic testing, among which the guidelines by the Dutch Pharmacogenetics Working Group (DPWG) and the Clinical Pharmacogenetics Implementation Consortium are internationally well recognized.^3,17,18,19^ To date, antidepressant treatment informed by these guidelines has not been compared with standard treatment in RCTs conducted in the clinical practice setting. Especially for tricyclic antidepressants (TCAs), pharmacogenetics may be of interest because therapeutic TCA plasma concentrations are well defined and treatment is frequently accompanied by a high burden of adverse effects.^11,20^ For attainment of a therapeutic TCA plasma concentration, multiple dosage adjustments over multiple weeks based on therapeutic drug monitoring are typically necessary.^12,21^ Meanwhile, suboptimal treatment can worsen depressive symptoms and suicidality, prolong treatment duration, and increase health-related costs.^22^

In the present study, we aimed to examine whether dosing based on pharmacogenetics according to the DPWG guidelines resulted in faster attainment of therapeutic TCA plasma concentrations compared with usual treatment. Furthermore, we investigated whether faster attainment of therapeutic concentrations was associated with higher effectiveness and fewer and less severe adverse effects. We hypothesized that application of the DPWG guidelines would result in faster attainment of therapeutic plasma concentrations, higher effectiveness, and a lower rate of adverse effects.

## Methods

### Study Design

The Pharmacogenetics for Individualized Tricyclic Antidepressant (PITA) dosing study was a multicenter RCT in which patients were enrolled between June 1, 2018, and January 1, 2022. The trial protocol is provided in Supplement 1. Ethical approval for this RCT was obtained from the Commissie Mensgebonden Onderzoek Medical Ethical Review Board in Arnhem-Nijmegen, the Netherlands. All inclusion sites obtained approval from their local ethical review boards. All patients provided written informed consent. This study followed the Consolidated Standards of Reporting Trials (CONSORT) reporting guideline for RCTs.

**Figure 1.**
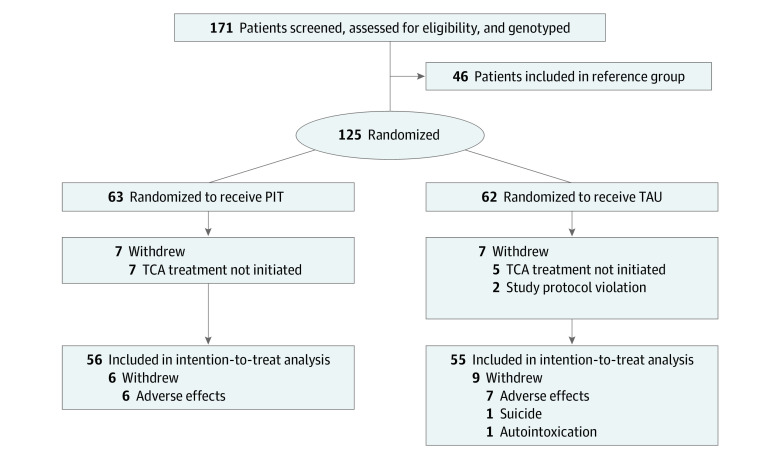
Flowchart of the PITA (Pharmacogenetics for Individualized Tricyclic Antidepressant [TCA]) Study The reference group consisted of nonrandomized patients with a cytochrome P450 2D6 isozyme (CYP2D6) normal metabolizer phenotype receiving usual treatment. PIT indicates pharmacogenetics-informed treatment; TAU, treatment as usual.

The participating institutions were hospitals and mental health care institutions in the Netherlands (eTable 1 in Supplement 2). All participants were tested for their *CYP2D6* and *CYP2C19* genotypes. Participants were subsequently randomized to receive PIT (PIT group) or usual treatment (control group) (Figure 1). Patients in the PIT group received a starting dosage based on their metabolizer phenotype according to the DPWG guidelines^22^ (eTables 2 and 3 in Supplement 2) whereas patients in the control group received the standard initial dosage (eTable 4 in Supplement 2). In both groups, TCA plasma concentrations were measured when a steady state plasma concentration was reached (ie, after 7 days without dosage adjustments).^11^ The clinical follow-up was 7 weeks.

### Participants

Patients were enrolled by their treating psychiatrist. They had a primary diagnosis of unipolar nonpsychotic MDD according to *Diagnostic and Statistical Manual of Mental Disorders* (Fifth Edition) criteria^23^ and were eligible for TCA treatment (nortriptyline, imipramine, or clomipramine) according to their psychiatrist. Diagnoses were established using the Structured Clinical Interview for *DSM-IV* Axis I Disorders.^24^ Additional inclusion criteria were aged 18 to 65 years and a 17-item Hamilton Rating Scale for Depression (HAMD-17) score of 19 or higher.^25^ Exclusion criteria were bipolar disorder, schizophrenia, or other primary psychotic disorder; substance use disorder in the past 3 months; intellectual disability; pregnancy or breastfeeding; a serious medical illness affecting the central nervous system; a contraindication for TCA use (eg, recent myocardial infarction); use of other drugs influencing the pharmacokinetics of TCAs (eg, CYP2D6 inhibitors); and use of psychotropic medications apart from benzodiazepines, which were allowed to a maximum of an equivalent of 4 mg of lorazepam per day. Data on race and ethnicity categories were not collected because the primary focus was on the interaction between CYP450 phenotypes and drug interactions. Most of the sample was of White race.

### Randomization

With the use of stratified block randomization, patients were randomized (1:1) to the PIT or control group by a staff member not involved in this study. The stratification was performed in the following order: (1) CYP2D6 metabolizer phenotype (and CYP2C19 metabolizer phenotype for those receiving imipramine), categorized as poor metabolizer (PM), intermediate metabolizer (IM), normal metabolizer (NM), and ultrarapid metabolizer (UM); (2) prescribed drug (nortriptyline, clomipramine, or imipramine); and (3) clinical setting (inpatient or outpatient), except for patients treated with imipramine who had a deviant metabolizer phenotype (PM, IM, or UM) for both CYP2D6 and CYP2C19 (eTable 3 in Supplement 2). Among those receiving imipramine with a deviant metabolizer phenotype for both CYP2D6 and CYP2C19, no pharmacogenetics-informed dosing advice was available in the DPWG guidelines, and patients were switched to nortriptyline and randomized accordingly.

In our original design, only patients with aberrant metabolizer phenotypes (PM, IM, or UM) were randomized to the PIT or control group, and patients with a CYP2D6 NM phenotype were assigned to the reference group. This reference group received the same treatment as the control group. After acceptance of a protocol amendment on November 25, 2019, all patients were randomized to the PIT or control group.

### Intervention

Patients randomized to the PIT group received an initial TCA dosage according to the DPWG guidelines (eTables 2 and 3 in Supplement 2).^17^ Patients randomized to the control group received a starting dosage based on the guideline by the Health Institute of the Netherlands (eTable 4 in Supplement 2).^26^ The treatment group allocation was not communicated to the patient or treating physician.

### Outcome Measures

The primary outcome was time (in days) to therapeutic TCA plasma concentrations. Secondary outcomes were severity of depressive symptoms and frequency and severity of adverse effects. Severity of depressive symptoms was measured weekly through the HAMD-17 (score range, 0-52, with higher scores indicating greater depression severity)^25^ by a blinded investigator. Adverse effects were scored weekly by the patients using a digital version of the Frequency, Intensity, and Burden of Side Effects Rating (FIBSER).^27^ The FIBSER consists of 3 items measuring frequency, severity, and burden of adverse effects, and each item (score range, 0-6, with higher scores indicating more severe interference with activities) was used as a secondary outcome measure.

### Genotyping and Therapeutic Drug Monitoring

*CYP2D6* and *CYP2C19* genotypes were assessed using DNA extracted from blood or saliva samples and measured according to the standard diagnostic flow in the participating institutes. The metabolizer phenotypes were established in accordance with Royal Dutch Pharmacists Association guidelines.^17^ Phenotypes were determined for alleles CYP2D6*1 to CYP2D6*11, CYP2D6*15, CYP2D6*17, CYP2D6*29, CYP2D6*35, CYP2D6*41, and duplications and for alleles CYP2C19*1 to CYP2C19*3 and CYP2C19*17.

When a steady state plasma concentration of TCA was reached, TCA plasma concentrations were measured using liquid chromatographic assays at the laboratories of the participating institutions and validated according to the guideline of the European Medicines Agency.^28^ In cases of subtherapeutic or supratherapeutic plasma concentrations, dosage adjustments were made based on linear kinetics until a therapeutic drug concentration was reached. Therapeutic plasma concentrations were defined as a nortriptyline concentration between 50 and 150 μg/L, a sum of clomipramine plus desmethylclomipramine concentration between 200 and 400 μg/L, or a sum of imipramine plus desipramine concentration between 150 and 300 μg/L.^26^

### Sample Size Calculation

To our knowledge, no previous studies have assessed time to attainment of a therapeutic TCA plasma concentration by pharmacogenetics-informed dosing according to DPWG guidelines. We assumed that 50% of the control group would reach a therapeutic plasma concentration within 4 weeks and that 50% of the PIT group would reach a therapeutic concentration within 2 weeks. Taking α = .05 and a power of 80% (2-sided log-rank test), a sample size of 44 patients per treatment group was required. For the secondary end points, we needed 63 patients per group (independent *t* test with 2-sided α = .05) after considering the mean reduction of adverse effects scores reported previously.^29^ To account for participant withdrawals before treatment initiation and unavailability for follow-up, we aimed to enroll 200 patients, resulting in 100 patients in each group.

### Statistical Analysis

All analyses were performed according to a modified intention-to-treat principle, meaning that patients were included if at least 1 TCA dose was administered. The primary analysis was presented in a Kaplan-Meier survival curve and conducted using a 2-sided log-rank test. In case no therapeutic plasma concentration was reached during the study, censoring was applied. We performed subgroup analyses per antidepressant (nortriptyline, clomipramine, and imipramine). Secondary outcome measures were analyzed through linear mixed-model analyses using a linear time trend (weeks of treatment) and the interaction term between study group (PIT or control) and time as an independent variable. We examined the interaction between treatment group and time for both depression severity (HAMD-17 score) and frequency and severity of adverse effects. Independent *t* tests were used to compare effectiveness and adverse effect scores among patients who completed the study (complete-case analysis). Regarding adverse effect scores, the highest reported score per item was analyzed. Statistical significance was defined as 2-sided *P* < .05. All analyses were performed using IBM SPSS Statistics, version 28 (IBM Corporation).^30^

## Results

### Baseline Characteristics

Among 171 included patients, 125 patients (73.1%) were randomized to the PIT (n = 63) or control (n = 62) group. A total of 14 randomized patients (11.2%) were excluded from the analysis because TCA treatment was not initiated due to early improvement of depressive symptoms or violation of the study protocol (Figure 1). Therefore, analyses were conducted for 111 patients (mean [SD] age, 41.7 [13.3] years; 69 [62.2%] female and 42 [37.8%] male); of those, 56 patients were in the PIT group and 55 were in the control group. The reference group consisted of 46 patients with a CYP2D6 NM phenotype.

At baseline, there were no differences between treatment groups with regard to sex, age, depression characteristics, CYP2D6 and CYP2C19 phenotype distribution, and type of TCA (Table). The majority of patients (107 [96.4%]) initiated TCA treatment with the advised dosage. Four patients (2 in the PIT group and 2 in the control group) started with a lower dosage than recommended. All patients attained the advised dosage within the first week of treatment. During the study period, 15 patients (13.5%) withdrew from the study. In the PIT group, 6 patients (10.7%) withdrew (2 patients after 1 week of treatment, 2 patients after 3 weeks, 1 patient after 4 weeks, and 1 patient after 6 weeks), all due to adverse effects of treatment. In the control group, 9 patients (16.4%) withdrew (3 patients after 1 week of treatment, 2 patients after 2 weeks, and 4 patients after 4 weeks); of those, 7 patients withdrew due to adverse effects of treatment, 1 due to suicide, and 1 due to autointoxication. For both groups, the dosing advice was well adhered to by the prescribers (96%).

**Table.** Baseline Characteristics of Patients

Characteristic	Patients, No. (%)^a^		
	Total (N = 111)	PIT group (n = 56)	Usual treatment group (n = 55)
Sex			
Female	69 (62.2)	36 (64.3)	33 (60.0)
Male	42 (37.8)	20 (35.7)	22 (40.0)
Age, mean (SD), y	41.7 (13.3)	40.8 (14.1)	42.7 (12.6)
HAMD-17 score, mean (SD)^b^	21.1 (4.8)	20.8 (4.7)	21.3 (4.9)
Duration of current depressive episode, y			
0-1	44 (39.6)	23 (41.1)	21 (38.2)
1-2	22 (19.8)	11 (19.6)	11 (20.0)
>2	45 (40.5)	22 (39.3)	23 (41.8)
Depressive episodes			
First	33 (29.7)	17 (30.4)	16 (29.1)
Recurrent	78 (70.3)	39 (69.6)	39 (70.9)
CYP2D6 phenotype			
PM	12 (10.8)	6 (10.7)	6 (10.9)
IM	57 (51.4)	30 (53.6)	27 (49.1)
NM	39 (35.1)	18 (32.1)	21 (38.2)
UM	3 (2.7)	2 (3.6)	1 (1.8)
CYP2C19 phenotype			
PM	3 (2.7)	2 (3.6)	1 (1.8)
IM	22 (20.0)	12 (21.4)	10 (18.2)
NM	80 (72.1)	39 (69.6)	41 (74.5)
UM	6 (5.4)	3 (5.4)	3 (5.5)
TCA			
Nortriptyline	67 (60.4)	34 (60.7)	33 (60.0)
Clomipramine	38 (34.2)	19 (33.9)	19 (34.5)
Imipramine	6 (5.4)	3 (5.4)	3 (5.5)

Abbreviations: CYP2C19, cytochrome P450 2C19 isozyme; CYP2D6, cytochrome P450 2D6 isozyme; HAMD-17, 17-item Hamilton Rating Scale for Depression; IM, intermediate metabolizer; NM, normal metabolizer; PIT, pharmacogenetics-informed treatment; PM, poor metabolizer; TCA, tricyclic antidepressant; UM, ultrarapid metabolizer.

^a^ Participants in the PIT and usual treatment groups were compared using χ^2^ tests or independent *t* tests, as appropriate.

^b^ Score range, 0-52, with higher scores indicating greater depression severity.

### Attainment of Therapeutic Plasma Concentrations

**Figure 2.**
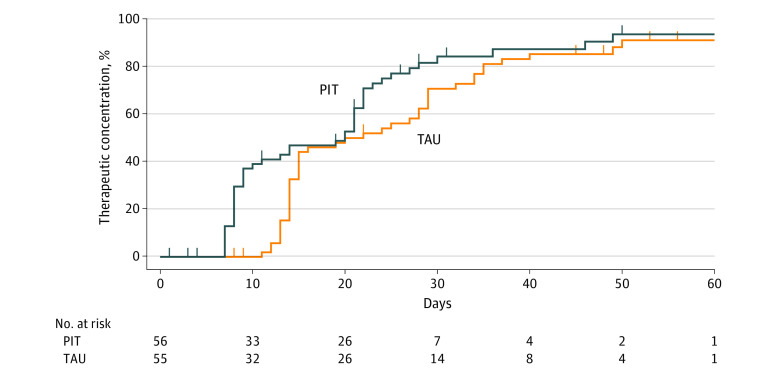
Survival Curves for Time to Therapeutic Plasma Concentrations of Tricyclic Antidepressants Overall Vertical bars on survival curves represent times of censoring. PIT indicates pharmacogenetics-informed treatment; TAU, treatment as usual.

A total of 47 patients (83.9%) in the PIT group and 45 patients (81.8%) in the control group attained a therapeutic concentration. The PIT group reached therapeutic concentrations significantly faster than the control group (mean [SD], 17.3 [11.2] days vs 22.0 [10.2] days; Kaplan-Meier χ^2^_1_ = 4.30; *P* = .04) (Figure 2). In the PIT group, 47 patients (83.9%) attained a therapeutic plasma concentration during the study period after a mean (SD) of 17.3 (11.2) days and a median of 14 days (range, 7-49 days). In the control group, 45 patients (81.8%) attained a therapeutic plasma concentration after a mean (SD) of 22.0 (10.2) days and a median of 16 days (range, 11-50 days). Mean plasma concentrations per CYP2D6 and CYP2C19 metabolizer phenotype are presented in eFigures 1 and 2 in Supplement 2, respectively.

**Figure 3.**
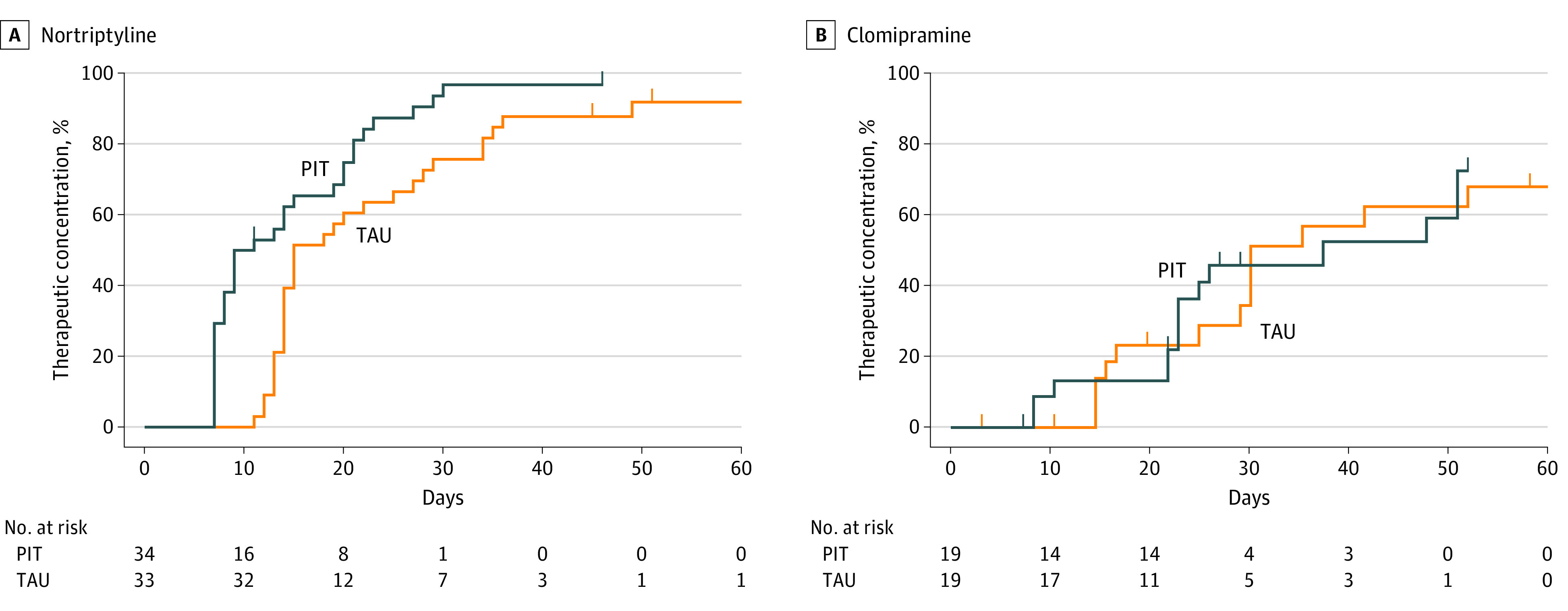
Survival Curves for Time to Therapeutic Plasma Concentrations of Nortriptyline and Clomipramine Vertical bars on survival curves represent times of censoring. PIT indicates pharmacogenetics-informed treatment; TAU, treatment as usual.

Post hoc analyses demonstrated that the effect was mainly found for nortriptyline (χ^2^_1_ = 9.30; *P* = .002; n = 67). No effect was observed for clomipramine (χ^2^_1_ = 0.23; *P* = .63; n = 38) and imipramine (χ^2^_1_ = 0.08; *P* = .78; n = 6) (Figure 3; the survival curve for imipramine is not shown due to the small number of patients [n = 6] receiving this TCA). Regarding nortriptyline, in the PIT group, 32 of 34 patients (94.1%) attained a therapeutic plasma concentration after a mean (SD) of 13.4 (7.4) days and a median of 9 days (range, 7-30 days) compared with 30 of 33 patients (90.9%) in the control group after a mean (SD) of 20.2 (9.6) days and a median of 15 days (range, 11-49 days). With regard to clomipramine, in the PIT group, 14 of 19 patients (73.7%) attained a therapeutic concentration after a mean (SD) of 25.9 (14.0) days and a median of 22 days (range, 8-49 days) compared with 13 of 19 patients (68.4%) in the control group after a mean (SD) of 25.8 (11.3) days and a median of 28 days (range, 14-50 days) (Figure 3).

### Effects on Depressive Symptoms

**Figure 4.**
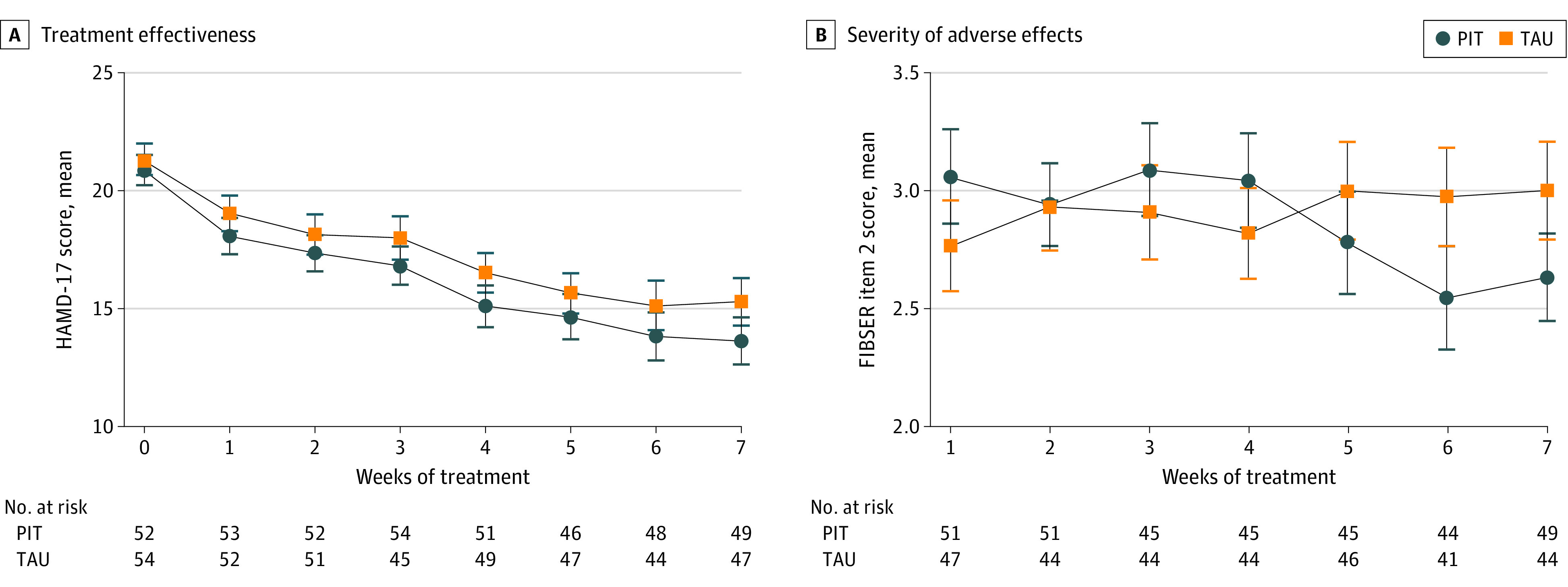
Treatment Effectiveness and Severity of Adverse Effects Treatment effectiveness was measured by the 17-item Hamilton Rating Scale for Depression (HAMD-17), and severity of adverse effects was measured by item 2 of the Frequency, Intensity, and Burden of Side Effects Rating (FIBSER). Whiskers represent SEs. PIT indicates pharmacogenetics-informed treatment; TAU, treatment as usual.

At baseline, depression severity (measured by HAMD-17 score) was similar between the PIT and control groups (Figure 4). After the first week until the last week of treatment, mean depression severity was lower in the PIT group compared with the control group. However, this observed difference between the PIT and control groups over time was not statistically significant (*F*_6,136_ = 0.45; *P* = .84). Post hoc analyses per specific TCA showed similar findings for nortriptyline and clomipramine (eFigure 3 in Supplement 2). A complete case analysis (n = 96) of the difference in HAMD-17 scores between baseline and after 7 weeks revealed no significant difference between the PIT and control groups (*t*_90_ = 0.66; *P* = .51; n = 92). Of the 96 patients who completed the study, 23 patients (24.0%) reached treatment response (defined as ≥50% reduction in the HAMD-17 score compared with baseline), and 16 patients (16.7%) attained remission (defined as a HAMD-17 score of <8 after 7 weeks of treatment).^31^

### Adverse Effects

The interaction between severity of adverse effects and time was significantly different between the PIT and control groups (*F*_6,114_ = 3.10; *P* = .008), whereby the severity of adverse effects decreased relatively more in the PIT group (Figure 4). By analyzing the highest reported value over the course of treatment, we found no significant difference between the PIT and control groups (*t*_105_ = 0.50; *P* = .62; n = 107). The results of post hoc analyses per specific TCA are shown in eFigure 3 in Supplement 2.

Interactions between the frequency of adverse effects (FIBSER item 1) and time (*F*_6,125_ = 4.03; *P* = .001) and between the burden of adverse effects (FIBSER item 3) and time (*F*_6,112_ = 2.56; *P* = .02) also differed significantly between the PIT and control groups (eFigure 4 in Supplement 2), which was consistent with our finding for the severity of adverse effects. A comparison of the highest reported value over the course of treatment demonstrated no significant difference between the PIT and control groups for the frequency (*t*_105_ = 1.26; *P* = .21; n = 107) and burden (*t*_105_ = 0.83; *P* = .41; n = 107) of adverse effects.

### Reference Group

In the reference group consisting of 46 nonrandomized patients with a CYP2D6 NM phenotype receiving usual treatment, 9 patients did not initiate TCAs; therefore, analyses were conducted for 37 patients. Baseline characteristics are shown in eTable 5 in Supplement 2. All patients received the advised initial dosage. During TCA treatment, 4 patients withdrew (1 patient after 1 week of treatment, 1 patient after 2 weeks, 1 patient after 5 weeks, and 1 patient after 6 weeks), all due to adverse effects of treatment. Of the 37 patients starting TCA therapy, 29 patients (78.4%) attained a therapeutic plasma concentration during the study period after a mean (SD) of 20.0 (11.2) days, which was comparable with patients who had a CYP2D6 NM phenotype in the control group (mean [SD], 20.6 [9.1] days; n = 16).

## Discussion

### Main Findings

In this first RCT to date comparing PIT with standard treatment, we found that PIT resulted in faster attainment of therapeutic TCA plasma concentrations without exposing patients to more severe adverse effects. Our analyses indicated a mean reduction of 5 days in time to attainment of therapeutic concentrations compared with usual treatment. This effect was primarily due to faster attainment of therapeutic nortriptyline concentrations, which showed a median reduction of 6 days compared with usual treatment. Based on these findings, we conclude that the DPWG guidelines can be effectively used to safely accelerate attainment of therapeutic TCA plasma concentrations.^17^

The difference between PIT and usual treatment can be explained by personalized initial dosages and bypassing of the buildup phase in the PIT group. The apparent difference between secondary (nortriptyline) and tertiary (clomipramine and imipramine) TCAs might be explained by metabolization pathways,^1,9,32^ although data on imipramine in our study were limited (n = 6). Nortriptyline metabolization mainly depends on CYP2D6, while other CYP450 enzymes (eg, CYP2C19, CYP3A4, and CYP1A2) are also involved for clomipramine.^1,9^ Dosing advice based on other CYP450 enzymes or a combination of deviant CYP450 enzymes is currently not incorporated in the DPWG guideline and was therefore not taken into account.

To date, only 1 study^33^ has been conducted in which CYP2D6-informed dosing was compared with standard treatment using therapeutic plasma concentrations as an outcome measure. In this study,^33^ nortriptyline and venlafaxine were investigated in older adults with MDD. For both antidepressants, the study did not detect a difference in the time needed to attain a therapeutic plasma concentration.^33^ Discrepant results compared with our study can be explained by a number of factors. First, the timing of the intervention was different. Clinicians only had access to pharmacogenetics-informed dosing advice at 14 days after treatment initiation,^33^ whereas in our study, this information could be used to determine the starting dosage. Second, the extent to which prescribers adhered to personalized dosing advice was questioned by the authors.^33^ In contrast, in our study, the dosing advice was well adhered to by the prescribers.

Regarding the secondary outcome measures, we found that faster attainment of therapeutic plasma concentrations did not translate into a significantly greater reduction in depressive symptoms or adverse effects. However, for adverse effects, we observed a different pattern in severity over time, suggesting that patients in the PIT group experienced gradually fewer and less severe adverse effects compared with those in the control group. In addition, it is clear that clinical outcome is influenced by many other biological and nonbiological factors in addition to antidepressant plasma concentrations.^5,34,35,36,37,38^

Most previous studies^39,40,41,42^ of treatment for MDD guided by pharmacogenetics examined the use of combinatorial pharmacogenetic tests (ie, multigene testing). Other studies^14,43,44,45^ found promising results regarding remission rates; however, questions have been raised about whether the studies were properly randomized and blinded.^43^ In most previous studies,^39,40,41,42^ information was not presented regarding how the pharmacogenetic test results were translated into the choice for a specific antidepressant or antidepressant dosage as well as the extent to which the prescribers adhered to the dosing advice in their treatment strategy. In contrast, our study design ensured that both prescribers and patients adhered well to the study protocol.

### Limitations

This study has several limitations. First, dosage adjustments based on therapeutic drug monitoring were performed weekly in both study groups; therefore, usual treatment was of higher quality than that found in standard clinical practice in which it takes several weeks until plasma concentrations are measured.^21^ This higher-quality treatment may result in an underestimation of the PIT effect compared with current clinical practice. Second, our sample size was relatively small; we included fewer patients than required for analyses on the secondary outcome measures and the follow-up duration was too short to draw definitive conclusions. Together with the clinical characteristics of patients in our study, who mainly had severe and chronic depression (which is associated with treatment nonresponse^46^), these factors might explain why we did not find a difference in treatment effectiveness between PIT and usual treatment. Third, we excluded patients using psychotropic medications and interacting concurrent medications; therefore, the results are not generalizable to all patients with MDD.

## Conclusions

This RCT found that application of the DPWG guidelines in TCA treatment of MDD could be safely applied and resulted in faster attainment of therapeutic plasma concentrations. No effect on depressive symptoms was found. The results of this study imply that the benefits of preemptive pharmacogenetic testing may vary between antidepressants. Therefore, further research that takes into account specific gene-antidepressant interactions with clinical outcomes is necessary.

## Notes

Supplement 1.
Trial Protocol

Supplement 2. **eTable 1.** Number of Patients per Inclusion Center, Presented as Numbers and Percentages **eTable 2.** Nortriptyline and Clomipramine Dosing Advice per CYP2D6 Metabolizer Phenotype According to DPWG Guidelines **eTable 3.** Imipramine Dosing Advice per CYP2D6 and CYP2C19 Metabolizer Phenotype According to DPWG Guidelines **eTable 4.** Standard Dosing Advice for Nortriptyline, Clomipramine, and Imipramine According to the Dutch Dosing Guideline (https://www.farmacotherapeutischkompas.nl/) **eFigure 1.** Comparison of TCA Plasma Concentrations Corresponding to PIT and TAU Dosing Advice per CYP2D6 Phenotype for (A) Nortriptyline, (B) Clomipramine, and (C) Imipramine, Presented as Tukey Plots and as Means With SDs **eFigure 2.** Comparison of TCA Plasma Concentrations in PIT and TAU Groups per CYP2C19 Phenotype for (A) Nortriptyline, (B) Clomipramine, and (C) Imipramine, Presented as Tukey Plots and as Means With SDs **eFigure 3.** Depression Severity (HAMD-17 Score) and Severity of Adverse Effects (FIBSER Item 2) for (A) Nortriptyline (n = 67) and (B) Clomipramine (n = 38), Presented as Means With SEs **eFigure 4.** Comparison Between PIT (n = 56) and TAU (n = 55) Regarding (A) Frequency (FIBSER Item 1) and (B) Burden of Adverse Effects (FIBSER Item 3), Presented as Means With SEs **eTable 5.** Baseline Characteristics of the Reference Group (n = 46) Compared With the PIT (n = 56) and TAU (n = 55) Groups

Supplement 3.
Data Sharing Statement
