# Supplementary material for: Effectiveness of Genotype-Specific Tricyclic Antidepressant Dosing in Patients With Major Depressive Disorder: A Randomized Clinical Trial
Source: JAMA Netw Open. 2023 May 8;6(5):e2312443. doi: 10.1001/jamanetworkopen.2023.12443 (PMC10167565; doi:10.1001/jamanetworkopen.2023.12443)
Supplement: Supplement 1. — Trial Protocol [file jamanetwopen-e2312443-s001.pdf]

RESEARCH PROTOCOL

Pharmacogenetics to improve personalized antidepressant dosing in patients with severe depression; a randomized controlled trial using Tricyclic Antidepressants

Version 5.0; 5 November 2019

PROTOCOL TITLE ‘Pharmacogenetics to improve personalized antidepressant dosing in patients with severe depression; a randomized controlled trial using Tricyclic Antidepressants’

**Protocol ID**	**NL63514.019.17**
**Short title**	**Personalized Tricyclic Antidepressant Dosing using pharmacogenetics.**
**Version**	**5.0**
**Date**	**5 November 2019**
**Coordinating investigator/project leader**	**J.G.E. Janzing Psychiatry Radboudumc PO Box 9101 6500 HB Nijmegen**<br>**Joost.Janzing@radboudumc.nl**
**Principal investigator(s) (in Dutch: hoofdonderzoeker/ uitvoerder)**<br><br>***<Multicenter research: per site>***	**J.G.E. Janzing Psychiatry Radboudumc PO Box 9101 6500 HB Nijmegen**<br>**Joost.Janzing@radboudumc.nl**<br><br>**Vincent van Gogh, Venray**<br>**Pro Persona (Nijmegen, Arnhem, Tiel and Ede)**<br>**Erasmus University Rotterdam dept. Psychiatry**<br>**Radboudumc Nijmegen dept. Psychiatry**<br>**Reinier van Arkel, ‘s Hertogenbosch**
**Sponsor**	**Radboudumc**
**Subsidising party**	**ZonMw ‘s Gravenhage**
**Independent expert (s)**	**Dr. C.C. Kan, Psychiatrist. Dept. Psychiatry Radboudumc PO Box 9101 6500 HB Nijmegen.**<br>**Tel. 024-3613489 e-mail: Cees.Kan@radboudumc.nl**

**Laboratory sites**	Department of Human Genetics Radboudumc<br>Internal mail 855 PO Box 9101 6500HB Nijmegen<br>&<br><br>Expertisecentrum Farmacogenetica Afdeling Klinische Chemie (Nb-415) Erasmus MC, Postbus 2040, 3000 CA Rotterdam<br><br>&<br><br>LAB Maastricht UMC+, Maastricht UMC, Postbus 5800, 6202 AZ MaastrichtThe above mentioned laboratories will perform the pharmacogenetics tests. Both labs take part in interlaboratory quality control (profiency testing) programs.<br><br>The assessments of TCA serum/plasma levels will be performed by the Pharmacy laboratories associated with the Mental Health Care institutions.<br><br>The Pharmacy laboratories also take part in interlaboratory quality control (proficiency testing) programs for measurement of TCAs. In addition all assays used for TCAs in plasma/serum at the Pharmacy laboratories will be crossvalidated.
**Pharmacy**	For outpatients regular medication will be delivered by the patient’s local pharmacy; in case of inpatients by the pharmacy associated with the hospital of admittance.

PROTOCOL SIGNATURE SHEET

Name	Signature	Date
**Sponsor or legal representative:**<br>***<please include name and function>***<br><br>***<For non-commercial research,>***<br>**Head of Department:**<br>***<include name and function>***	**Prof. dr. A.H. Schene**<br>**Head dept of Psychiatry**<br>**Radboudumc Nijmegen**	
**[Coordinating Investigator/Project leader/Principal Investigator]:**<br>***<please include name and function>***	**Dr. J.G.E. Janzing**<br>**Psychiatrist**	

TABLE OF CONTENTS

1. INTRODUCTION AND RATIONALE 10
OBJECTIVES 11
2. STUDY DESIGN 12
STUDY POPULATION 13
2.1 Population (base) 13
2.2 Inclusion criteria 13
2.3 Exclusion criteria 14
2.4 Sample size calculation 14
3. TREATMENT OF SUBJECTS 15
3.1 Investigational product/treatment 16
3.2 Use of co-intervention (if applicable) 16
3.3 Escape medication (if applicable) 16
4. INVESTIGATIONAL PRODUCT 17
This section is not applicable. 17
4.1 Name and description of investigational product(s) 17
4.2 Summary of findings from non-clinical studies 17
4.3 Summary of findings from clinical studies 17
4.4 Summary of known and potential risks and benefits 17
4.5 Description and justification of route of administration and dosage 17
4.6 Dosages, dosage modifications and method of administration 17
4.7 Preparation and labelling of Investigational Medicinal Product 17
4.8 Drug accountability 17
5. NON-INVESTIGATIONAL PRODUCT 18
5.1 Name and description of non-investigational product(s) 18
5.2 Summary of findings from non-clinical studies 18
5.3 Summary of findings from clinical studies 18
5.4 Summary of known and potential risks and benefits 18
5.5 Description and justification of route of administration and dosage 18
5.6 Dosages, dosage modifications and method of administration 18
5.7 Preparation and labelling of Non Investigational Medicinal Product 18
5.8 Drug accountability 18
6. METHODS 19
6.1 Study parameters/endpoints OUTCOME PARAMETERS: 19
6.1.1 Main study parameter/endpoint 19
6.1.2 Secondary study parameters/endpoints (if applicable) 19
6.1.3 Other study parameters (if applicable) 20
6.2 Randomisation, blinding and treatment allocation 20
6.3 Study procedures 20
6.4 Withdrawal of individual subjects 23
6.4.1 Specific criteria for withdrawal (if applicable) 23
6.5 Replacement of individual subjects after withdrawal 24

6.6 Follow-up of subjects withdrawn from treatment 24
6.7 Premature termination of the study not applicable. 24
7. SAFETY REPORTING 25
7.1 Temporary halt for reasons of subject safety 25
7.2 AEs, SAEs and SUSARs 25
7.2.1 Adverse events (AEs) 25
7.2.2 Serious adverse events (SAEs) 25
Suspected unexpected serious adverse reactions (SUSARs): not applicable 26
7.3 Annual safety report 26
not applicable 26
7.4 Follow-up of adverse events 26
7.5 [Data Safety Monitoring Board (DSMB) / Safety Committee] 26
8. STATISTICAL ANALYSIS 27
8.1 Primary study parameter(s) 27
8.2 Secondary study parameter(s) 27
8.3 Other study parameters 27
8.4 Interim analysis (if applicable) 28
Not applicable 28
9. ETHICAL CONSIDERATIONS 28
9.1 Regulation statement 28
9.2 Recruitment and consent 28
9.3 Objection by minors or incapacitated subjects (if applicable) 28
9.4 Benefits and risks assessment, group relatedness 28
9.5 Compensation for injury 28
9.6 Incentives (if applicable) 29
ADMINISTRATIVE ASPECTS, MONITORING AND PUBLICATION 29
9.7 Handling and storage of data and documents 29
9.8 Monitoring and Quality Assurance 30
9.9 Annual progress report 30
9.10 Temporary halt and (prematurely) end of study report 30
10. Public disclosure and publication policy 31
11.1 Synthesis 33
12. REFERENCES 33

LIST OF ABBREVIATIONS AND RELEVANT DEFINITIONS

**ABR**	**ABR form, General Assessment and Registration form, is the application form that is required for submission to the accredited Ethics Committee (In Dutch, ABR = Algemene Beoordeling en Registratie)**
**AE**	**Adverse Event**
**AR**	**Adverse Reaction**
**CA**	**Competent Authority**
**CCMO**	**Central Committee on Research Involving Human Subjects; in Dutch: Centrale Commissie Mensgebonden Onderzoek**
**CV**	**Curriculum Vitae**
**DSMB**	**Data Safety Monitoring Board**
**EU**	**European Union**
**EudraCT**	**European drug regulatory affairs Clinical Trials**
**GCP**	**Good Clinical Practice**
**IB**	**Investigator's Brochure**
**IC**	**Informed Consent**
**IMP**	**Investigational Medicinal Product**
**IMPD**	**Investigational Medicinal Product Dossier**
**METC**	**Medical research ethics committee (MREC); in Dutch: medisch ethische toetsing commissie (METC)**
**(S)AE**	**(Serious) Adverse Event**
**SPC**	**Summary of Product Characteristics (in Dutch: officiële productinfomatie IB1-tekst)**
**Sponsor**	**The sponsor is the party that commissions the organisation or performance of the research, for example a pharmaceutical company, academic hospital, scientific organisation or investigator. A party that provides funding for a study but does not commission it is not regarded as the sponsor, but referred to as a subsidising party.**
**SUSAR**	**Suspected Unexpected Serious Adverse Reaction**
**Wbp**	**Personal Data Protection Act (in Dutch: Wet Bescherming Persoonsgevens)**
**WMO**	**Medical Research Involving Human Subjects Act (in Dutch: Wet Medisch-wetenschappelijk Onderzoek met Mensen**

SUMMARY

Rationale: Tricyclic Antidepressants (TCAs) are the cornerstone of treatment for patients with severe Major Depressive Disorder (sMDD). Current dosing is guided by repeated measurements of serum/plasma levels. Compared to patients with a normal metabolization function, for those with increased cytochrome P450 (CYP450) enzyme activity it takes longer to reach a therapeutic drug level. As a consequence patients have a prolonged treatment period, increased risk of suicidal behaviour and eventually lower remission rates. For those with reduced CYP450 activity higher rates of side effects are expected. Another TCA dosing strategy, taking the genetic variants of the *CYP2D6* and *CYP2C19* genes into account, may help to reduce the above mentioned problems. Guidelines are available for this alternative dosing strategy and it is being used in selected centres and by individual psychiatrists in The Netherlands. In this implementation research project we hypothesize that genotype informed dosing results in faster attainment of therapeutic drug levels, lower rates of side effects, earlier symptom relief and lower levels of health- and working related costs

Objective:

Primary Objective:

To assess whether CYP450 genotype guided dosing of TCAs results in faster attainment of therapeutic plasma concentrations compared to dosing as usual. Secondary Objectives:

- To assess whether genotype guided dosing results in lower rates of adverse effects compared to dosing as usual. .
- To assess whether genotype guided dosing results in earlier reductions of depressive symptoms compared to dosing as usual. .
- To assess the value of an early TCA serum/plasma level (12 hours after the first dose) as a potential predictor of time to therapeutic plasma concentration, treatment response and side effects.
- To explore the contribution of other clinical and biological factors and changes within these factors during treatment as predictors of time to therapeutic plasma concentration, treatment response and side effects.
- To compare the healthcare costs and work related costs of genotype based dosing to dosing as usual. .

Study design: This study is a randomized controlled clinical trial. As we aim to approach actual clinical practice (which is important for generalisation and implementation of the results), prescribing physicians will be unblinded for the *CYP2C9* and *CYP2D6* genotype and the resulting metabolization phenotype. This will not affect the attainment of therapeutic serum/plasma concentrations as primary endpoint in this study, as we are using a predetermined dosing algorithm and the endpoint is an objective laboratory measurement.

Study population: Patients are in- and outpatients, having a primary diagnosis of severe major depressive disorder (SCID-I diagnosis in agreement with DSM-5 criteria and a Hamilton Rating Scale for Depression score ≥ 19 (HAM-D 17-item version), aged 18-65 years, who, according to their physician, are eligible for treatment with a TCA (Nortriptyline, Clomipramine or Imipramine). The choice of the specific TCA is at the discretion of the physician in attendance.

Intervention (if applicable):

Using a genetic test, genetic variants that have an impact on the metabolizing capacity of CYP2D6 and CYP2C19 will be determined. Based on the results of this test, patients will be classified into a metabolic phenotype category (PM,IM,EM or UM) and can thus be given a genotyped-guided dose (according to the KNMP dosing guidelines). A total of 200 patients will be randomized in two strategies: 100 patients will receive TCA dosing according to existing pharmacogenetic dosing guidelines (KNMP) and 100 patients will receive TCA dosing according to the conventional dosing guideline (Farmacotherapeutisch Kompas).

Main study parameters/endpoints: Primary outcome measure: Time to TCA plasma concentration in the therapeutic range (primary outcome measure).

Nature and extent of the burden and risks associated with participation, benefit and group relatedness:

Overall, there will be a maximum of 4 extra blood withdrawals: one for the CYP450 genotyping, two samples for plasma storage (one at baseline and one after 7 weeks of treatment) and one for the 12 hours TCA serum/plasma level analysis at day 1 of treatment. The first sample will be taken together with the regular blood withdrawal for standard laboratory investigations. For the other samples extra venepunctures are necessary. If a patient prefers not to give blood for genetic testing we will isolate DNA from the saliva. In addition, the assessments necessary for cost effectivity analysis will give extra burden (beyond standard high care). We do not expect that patients in this study are exposed to extra risk besides the risks of venepuncture. Instead we expect lower risk levels: in the intervention group the TCA doses are adjusted to the individual CYP450 phenotype based on the most actual KNMP guidelines and therefore we expect lower levels of side effects and earlier treatment response. The weekly monitoring for side effects and depressive symptoms in all participating patients give a low burden to the patients but contribute to extra safety compared to regular care. Our study is aimed at decreasing risk in the patients by lowering the chances for side effects and reducing the time to therapeutic response compared to treatment as usual.

1. INTRODUCTION AND RATIONALE

Major Depressive Disorder (MDD) is a severe psychiatric condition characterized by episodes of low mood, anhedonia and impaired functioning. The life time prevalence is 16-20% and it is accompanied by a large burden due to health related costs, work impairment, a severe reduction of quality of life and an increased risk for suicidal behaviour (Kessler & Bromet 2013).

Antidepressant pharmacotherapy is the cornerstone for treatment of severe depression. Unfortunately, treatment results are disappointing and successful antidepressant treatment often requires multiple attempts with different drug types or drug combinations (Rush et al. 2006). Indeed, a recent comment emphasized that the matching between patients and treatments requires a prolonged period of “trial and error”, delaying clinical improvement and increasing the risk and costs associated with treatment (Chekroud & Krystal 2015).

Personalized treatment, taking individual patient characteristics into account may address this problem and has therefore been selected as one of main targets of the recently developed Dutch Research Agenda for Mental Health (Onderzoeksagenda GGz 2016). According to current guidelines (MRD 2013; APA 2010) Tricyclic Antidepressants (TCAs) pharmacotherapy is the treatment of choice for severe depression, including depression in hospitalized patients. An important advantage of TCAs compared to other groups of antidepressants is that therapeutic reference ranges have been established (Hiemke et al. 2011). For plasma or serum concentrations within these ranges an optimal ratio between drug efficacy and adverse effects has been demonstrated. In clinical practice it is critically important to reach the therapeutic window as fast as possible.
According to current guidelines, a relatively low starting dose is prescribed and repeated measurements of plasma levels of drug and active metabolite (Therapeutic Drug Monitoring, TDM) are necessary to guide and individualize the dose of TCA. Unfortunately, large inter-individual differences exist in the relationship between TCA dose and plasma levels. For patients with *increased* activity of specific cytochrome P450 (CYP) isoenzymes (Ultrarapid Metabolizers, UM) dosing as usual is inefficient as initial TCA concentrations tend to be low and it takes several weeks to reach the therapeutic window. The subsequent delay is associated with enduring personal suffering, increased numbers of hospital days, increased risk for suicidal behaviour and eventually lower recovery rates for depression. Patients with *decreased* CYP450 activity (Intermediate Metabolizers (IM) and Poor Metabolizers (PM)) have relatively high systemic exposure to TCAs. Traditional dose finding may be accompanied by the occurrence of increased rates of adverse effects. These adverse effects

are inconvenient (dry mouth, sedation) but may also be dangerous (confusion, respiratory depression and cardiac arrhythmia).

Inter-individual differences in activity of CYP450 isoenzymes are determined by genetic variants (polymorphisms) in the associated *CYP* genes. The Pharmacogenetics Working Group of the Royal Dutch Association for the Advancement of Pharmacy (KNMP) and the international Clinical Pharmacogenetics Implementation Consortium (CPIC) published dosing strategies for TCAs based on genetic variants in *CYP2D6* and *CYP2C19* (Swen et al. 2011; https://kennisbank.knmp.nl; Hicks et al. 2016). To this end, randomized trials focussing on effectiveness, prevention of adverse effects and cost-effectiveness of genotype based dosing in larger groups of patients are not available from the literature (Hicks et al. 2016).

Therefore, the added value of pharmacogenetics is still unknown. Information on (cost)effectiveness is essential before further implementation can be decided on and genotype based dosing can be included in psychiatric prescription guidelines.

The overall aim of the present study is to provide the evidence base for pharmacogenetics based dosing of TCAs in patients with severe MDD. To this end we will make a direct comparison between (a) genotype-based dosing (according to the Dutch guidelines of the KNMP) supplemented with Therapeutic Drug Monitoring (TDM) and (b) dosing as usual supplemented with TDM. We hypothesize that personalized genotype-guided dosing of TCAs results in faster achievement of therapeutic serum/plasma levels and consequently earlier reductions of depressive symptoms and reductions of adverse effects and reductions in healthcare costs and costs related to work impairment compared to dosing as usual.

OBJECTIVES

Primary Objective:
To assess whether CYP450 genotype-guided dosing of TCAs results in faster attainment of therapeutic plasma concentrations compared to dosing as usual..

Secondary Objectives:

- To assess whether genotype guided dosing results in lower rates of adverse effects compared to dosing as usual. .
- To assess whether genotype guided dosing results in earlier reductions of depressive symptoms compared to dosing as usual. .
- To assess the value of an early TCA serum/plasma level (12 hours after the first dose) as a potential predictor of time to therapeutic plasma concentration, treatment response and side effects.

- To explore the predictive value of other clinical (psychomotor retardation) and biological factors (measures of metabolomics) and the changes within these factors during treatment to therapeutic plasma concentration, treatment response and side effects.
- To compare the healthcare costs and work related costs of genotype based dosing to dosing as usual.

2. STUDY DESIGN

DESIGN CLINICAL TRIAL This study is a multi-center randomized controlled clinical trial. As we aim to approach actual clinical practice (which is important for generalization and implementation of the results), prescribing physicians will be unblinded for the genotype and the resulting metabolization phenotype. Outcome assessments will be performed by researchers who are blinded for group assignment (clinical assessment scales) and the patients themselves (self-assessment scales). The pragmatic design of this study is similar to the design of other pharmacogenetics studies (e.g. Berm et al. 2015 Coenen et al. 2015).
As the results of this study are aimed to be generalized to current clinical practice we will perform the study in different centers (multi-center study). The dosing instructions and the assessments will be strictly monitored to increase comparability between groups. If protocols tend to be violated we will also perform a per protocol analysis. The primary outcome measure is based on a laboratory value. The assessments of side effects, depressive symptoms, quality of life and costs are self-rated by the patients or by members of the research team (research physician, research nurse) but not the treating clinician. For details of the administration of the scales: see the section on the research instruments.

1. Phase 1. In the screening phase all patients fulfilling inclusion criteria (see below) will be genotyped for genetic variants in the *CYP2C19* and *CYP2D6* genes.
2. Phase 2. All subjects will be randomized to one of the strategies below.

The patient will be followed for a period of 26 weeks. This part of the study comprises the analysis of the primary and secondary (including the cost effectiveness study) endpoints (most of them assessed after 7 weeks).

The figure below shows the aimed inclusion process.

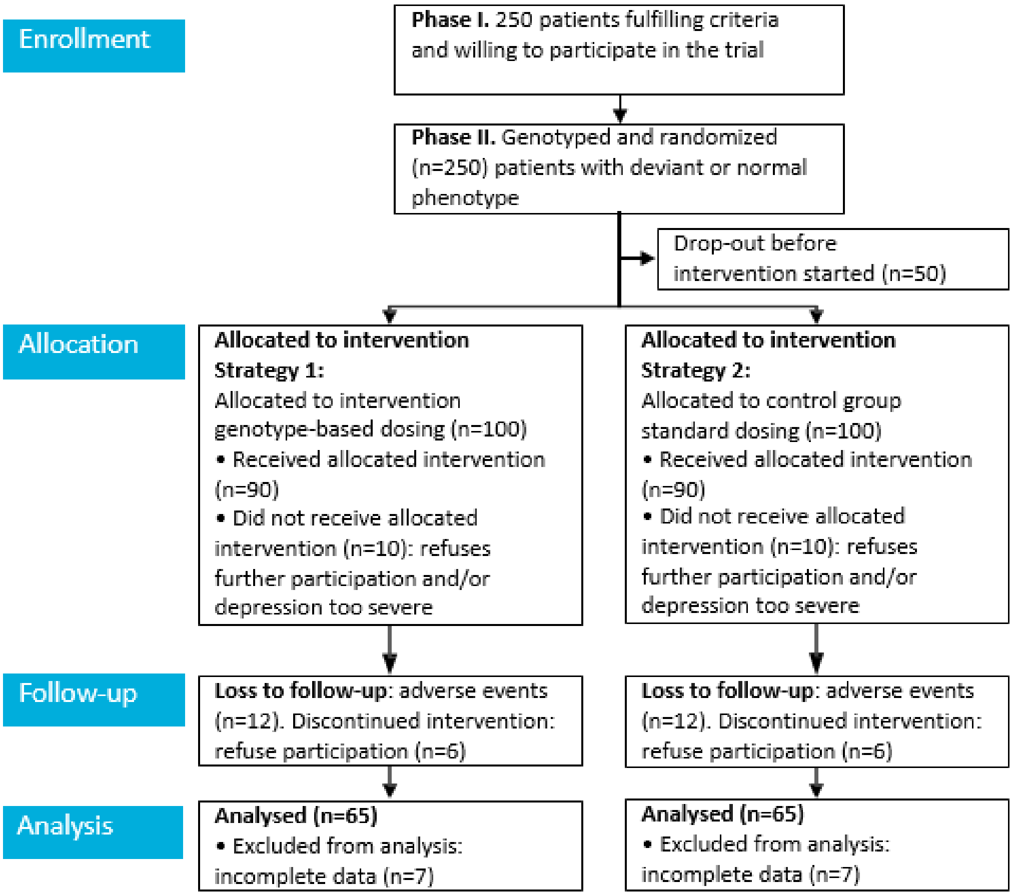

STUDY POPULATION

2.1 Population (base)

Patients will be recruited at the in- and outpatient departments of the 7 following centres: department of Psychiatry at Radboudumc Nijmegen, Pro Persona (TOP-GGZ) Expertise Centre for Depression , Pro Persona Location Tarweweg, Nijmegen, Nijmegen, Pro Persona Location Arnhem, Pro Persona Location Ede, Pro persona Location Tiel, Vincent van Gogh (TOP-GGZ) Expertise Centre for Neuropsychiatry, Venray, Vincent van Gogh Venlo, Reinier van Arkel 's-Hertogenbosch, Elisabeth-TweeSteden Tilburg and the Department of Psychiatry Erasmus MC Rotterdam. Patients will be screened for inclusion criteria and informed about the study by their physician. Patients are in- and outpatients with a diagnosis of severe major depressive disorder for whom, according to their treating physician, TCA treatment is indicated. Details about the estimated numbers of patients and estimated inclusion rates per participating centre can be found in the appendix of the original Application form ZonMw GGG file number 80-84800-98-16001 which will be uploaded.

2.2 Inclusion criteria

In order to be eligible to participate in this study, a subject must meet all of the following criteria: Patients are in- and outpatients, having a primary diagnosis of severe major

depressive disorder (SCID-I diagnosis in agreement with DSM-5 criteria and a Hamilton Rating Scale for Depression score ≥ 19 (HAM-D-17-item version; Hamilton 1960), aged 18-65 years, who, according to their physician, are eligible for treatment with a TCA (Nortriptyline (NOR), Clomipramine (CLOMI) or Imipramine (IMI)). The choice of the specific TCA is at the discretion of the physician in attendance.

2.3 Exclusion criteria

A potential subject who meets any of the following criteria will be excluded from participation in this study: (1) Psychotic depression (2) Bipolar I or II disorder (3) Schizophrenia or other primary psychotic disorder (4) Drug or alcohol dependence in the past 3 months. (5) Mental Retardation (IQ < 80). (6) For women: pregnancy or possibility for pregnancy without adequate contraceptive measures (questioned by physician). (7) Breast-feeding. (8) Serious medical illness affecting the CNS, including but not restricted to M Parkinson, SLE, brain tumour, CVA. (9) Relevant medical illness as contra-indication for TCA use, such as recent myocardial infarction. (10) Other drugs influencing the pharmacokinetics of the TCAs as based on an up to date list (derived from Micromedex www.micromedexsolutions.com). In case of psychotropic co-medication only a benzodiazepine in a dose equivalent up to 4 mg lorazepam will be allowed.

2.4 Sample size calculation

SAMPLE SIZE CALCULATION
Based on clinical guidelines and experience, we designed a survival model according to which 50% (median survival) of the control group reaches steady state concentrations within the therapeutic range at 4 weeks. We assume that 50% of the intervention group will reach steady state concentrations within the therapeutic range at a maximum of 2 weeks. This difference of only 2 weeks is a conservative estimate as patients in the intervention group may immediately start at an optimal dose based on pharmacogenetic testing results. Taking an alpha level of 5%, two-sided tests of significance and a power of 80%, the model predicts a sample size of 44 patients per group. An essential condition imposed by the subsidising party (ZonMW) was that the sample size should be enough to answer two of the secondary research questions (regarding depression scores and side effects) as well. For both of the secondary measures we need 63 patients per group based on a reduction of the mean level of side effects (Frequency, Intensity, and Burden of Side Effects Rating FIBSER (Wisniewski et al. 2006)) from 2.98 (sd=1.88) to 2.04 (based on Star-d study results) For depression scores we expect a mean HAM-D reduction in the intervention group of 17.5 (sd=9) points compared to 13 points in controls (adapted from the DUDG study Wijkstra et al. 2010). To account for drop-out before treatment initiation and loss to follow-up we aim to include 250 patients, resulting in 100 patients in each arm

The sample size calculation for this study was performed by a statistician of the Department of Health Evidence, Radboudumc (dr R. Donders).

3. TREATMENT OF SUBJECTS

INTERVENTION

Genotyping: For eligible patients genetic variants that affect the metabolising capacity of CYP2D6 and CYP2C19 will be determined. These variants explain in 90-95% of the cases with changes in CYP2D6 and CYP2C19 enzyme activity (see Appendix A). Dedicated genotyping methods will be used for the detection of the relevant genetic variants. Both labs that will perform genotyping take part in interlaboratory quality control programs.

Based on the drug choice and based on the latest allele definition tables (as present in the KNMP kennisbank achtergrondteksten) each patient will be classified into a metabolic phenotype category (PM,IM,EM or UM; see Appendix B).

Strategy 1: Genotype-guided dosing with Therapeutic Drug Monitoring (100 patients). In these patients, initial dosing of the TCAs will take place according to the pharmacogenetic dosing guidelines by the KNMP (https://kennisbank.knmp.nl; see Appendix C). These guidelines recommend specific dose adaptations for each of the metabolizer genotypes and for each type of TCA. The initial dose advice is communicated to the treating physician. In agreement with common practice, each time a steady-state can be expected (7 days after the last dose change) plasma concentrations of TCA and active metabolites will be measured. If the predefined therapeutic level has not been reached yet, further dose adaptations will be communicated, based on a predetermined dosing algorithm that considers linear pharmacokinetics of TCAs. This procedure will be repeated until a therapeutic level at steady state has been reached (endpoint of primary outcome).

Strategy 2: Standard dosing with Therapeutic Drug Monitoring (100 patients). In these patients, initial dosing of TCA will be performed according to standard Dutch dosing guidelines (Farmacotherapeutisch Kompas). According to these guidelines the recommended starting dose per drug is given which is gradually increased during the first week until the standard dose is reached. When steady-state has been achieved for the standard dose (7 days later), plasma concentrations will be measured and dosing will be further adjusted using the same dosing algorithm based on linear kinetics (Birkenhäger and Moleman 2007) until a therapeutic level at steady state has been reached (as described under strategy 1).

In additional analyses we will compare the results of the deviant metabolizer groups with those of patients with this normal metabolization phenotypeBefore inclusion, all patients will have signed the informed consent form.

3.1 Investigational product/treatment

Not applicable.

3.2 Use of co-intervention (if applicable)

Not applicable

3.3 Escape medication (if applicable)

Not applicable

4. INVESTIGATIONAL PRODUCT

This section is not applicable.

4.1 Name and description of investigational product(s)

4.2 Summary of findings from non-clinical studies

4.3 Summary of findings from clinical studies

4.4 Summary of known and potential risks and benefits

4.5 Description and justification of route of administration and dosage

4.6 Dosages, dosage modifications and method of administration

4.7 Preparation and labelling of Investigational Medicinal Product

4.8 Drug accountability

5. NON-INVESTIGATIONAL PRODUCT

This section is not applicable. The drugs used for treatment in this study (imipramine, clomipramine and nortriptyline) are regularly prescribed and registered drugs. For product details we refer to the Summary of Product Characteristics (SPC) at the CBG website **https://db.cbg-meb.nl**.

5.1 Name and description of non-investigational product(s)

5.2 Summary of findings from non-clinical studies

5.3 Summary of findings from clinical studies

5.4 Summary of known and potential risks and benefits

5.5 Description and justification of route of administration and dosage

5.6 Dosages, dosage modifications and method of administration

5.7 Preparation and labelling of Non Investigational Medicinal Product

5.8 Drug accountability

6. METHODS

6.1 Study parameters/endpoints OUTCOME PARAMETERS:

6.1.1 Main study parameter/endpoint

1. Primary outcome measure: Time to TCA plasma concentration in the therapeutic range (primary outcome measure). Serum/plasma for plasma concentration measurements will be taken in the morning, at 10-16 hours after the last evening dose when prescribed once daily. For patients with a two or three daily regime, plasma concentration measurement will be taken in the morning before morning dose. Concentrations will be measured using validated liquid chromatography (LC) assays, to be performed by the clinical pharmaceutical laboratories associated with the centres where patients are included. Assays for TCAs performed well in interlaboratory quality control (proficiency testing) programs and will be cross validated. TCA plasma concentration assessments are performed at steady state (after 7 days of a stable dose) and as described before, dose adaptations are based on these measurements using a predetermined dosing algorithm. When a patient attains a plasma level within the therapeutic range at steady state, the primary endpoint has been reached (for nortriptyline 0.05–0.15 mg/l; for clomipramine: 0.2–0.30 mg/l (clomipramine plus desmethylclomipramine) and for imipramine 0.15-0.3 mg/l (imipramine plus desipramine).

6.1.2 Secondary study parameters/endpoints (if applicable)

a. **Reduction of depressive symptoms after 7 weeks of treatment** (defined as the baseline HAM-D score (Hamilton 1960) minus the HAM-D score at the 7 weeks assessment.) rated by a blinded investigator and the reduction on the QIDS-SR (Rush et al. 1986) rated by the patients themselves.
b. **Highest level of side effects** based on the Antidepressant Side Effect Checklist (ASEC, Uher et al. 2009) and the FIBSER (Frequency, Intensity, and Burden of Side Effects Rating FIBSER (Wisniewski et al. 2006)) rated by the patient.
c. **Economic Evaluation**:
The impact of the intervention on the quality of life of patients will be assessed both by the EuroQol 5 dimensions with 5 levels (EQ5D5L; Lamers et al. 2005) and the The Short Form (36) Health Survey (SF36; RUG 1993) at weeks 0, 2, 4, 6, 13 and 26 following randomization.

Cost analysis: The cost analysis consists of two main parts. First, at patient level, volumes of care related to MDD and TCA therapy will be measured by means of the iMTA Medical Consumption Questionnaire. This questionnaire measures all relevant health care related costs like outpatient visits at any medical specialist and hospitalizations. In addition the medication use will be derived from the electronic patient records. Loss of productivity due to illness or recovery, will be estimated based on patient reported absences from paid (or unpaid) labour measured with the Productivity Cost Questionnaire.

The second part of the cost analysis consists of determining the cost prices for each volume of consumption. The standard cost prices from the 'Dutch Guidelines for Cost Analyses' and www.medicijnkosten.nl will be used. For units of care where no standard prices are available real costs prices will be determined on the basis of full cost pricing. Productivity losses will be valued by means of the friction cost method. In the end volumes of care will be multiplied with the cost prices for each volume of care to calculate costs.
For more detailed information regarding the economic evaluation see the study description below (7.3).

6.1.3 Other study parameters (if applicable)

Not applicable

6.2 Randomisation, blinding and treatment allocation

Randomization: 200 patients will be 1:1 randomized over the two different dosing strategies. We will use stratified block randomisation using the computer programme CASTOR (nl.castoredc.com). In order to make comparable groups, the stratification will take 1. CYP-based phenotype, 2. drug type (NOR vs.CLOMI vs.IMI), and 3. baseline patient status (inpatient vs. outpatient) into account (in this order). An exception to this randomization protocol is when a patient carries a deviant cytochrome P450 metabolism for both CYP2D6 and CYP2C19 while imipramine is prescribed. In this case, patients will be switched to nortriptyline and randomized accordingly. As the number of stratification variables should be limited to avoid empty cells, we will plan additional statistical adjustments for gender, age and sociocultural differences in case of imbalance between study groups.

6.3 Study procedures

The table below shows the procedures according to the research proposal.

**Visit**<br>**day**<br>**week**<br>**Procedure**	1<br>-7<br>-1	2<br>0<br>0	3<br>7<br>1	4<br>14<br>2	5<br>21<br>3	6<br>28<br>4	7<br>35<br>5	8<br>42<br>6	9<br>49<br>7
Informed consent	x								
SCID-I	x								
Psychiatric assessments	x								
Medical history	x								
CYP 450 genotype	x								
Laboratories + ECG	x								
Plasma for storage		x							x
Physical examination	x								
SRRS-6 (retardation)		x							x
Medication prescribed (TCA)		x	x	x	x	x	x	x	x
Plasma levels	12 hours after first dose and repeated at steady state until primary endpoint has been reached								
Adverse effects (ASEC)		x	x	x	x	x	x	x	x
HAM-D/QIDS	x	x	x	x	x	x	x	x	x
EQ5D and SF36		x		x		x		x	

Description:
To maximize generalization of the results we aim to perform the study according to the principles of high standard care for severely depressed patients for whom a tricyclic antidepressant is prescribed. Therefore the baseline investigations include a formal psychiatric assessment which includes SCID-I (Structured Clinical Interview for DSM-IV Axis I: a clinical interview designed to elicit the DSM-IV criteria). Based on the SCID-I we conclude if patients fulfil psychiatric inclusion criteria. Other routine procedures in clinical practice are assessments of Medical history, physical examination and Laboratory investigations (Hb, Ht, Ery's, Leuco's, Diff, Na, K, Ureum, Creat, ASAT, ALAT, LDH, yGTP, Free T4, TSH) and ECG (only when indicated), examination physical examination are all in agreement with the procedures that should be offered in high standard clinical care and cannot considered to be a extra burden to the patients. Based on these investigations we can decide if patients fulfil inclusion criteria.

Repeated administration of scales for the measurements of the depressive symptoms (HAM-D/QIDS), adverse events (ASEC and FIBSER) and the measurements of TCA plasma levels are also in agreement with careful common clinical practice.

To answer the main research question, CYP450 genotyping will take place (blood withdrawal will take place together with that for laboratory investigations). At baseline and after 7 weeks we will take plasma for storage and the SRRS-6 retardation scale (Widlöcher et al. 1989; observational scale scored by the researcher), will be administrated. Twelve hours after the first TCA dose an early plasma TCA level will be measured. Other interventions are administration of the assessment instruments for cost effectivity analysis (see below).

Study procedure:

Visit 1 (Inclusion and genotyping): All eligible patients will be asked to provide written informed consent. Inclusion and exclusion criteria are checked and baseline and demographic variables will be collected (complete somatic and psychiatric history, physical examination, inventory of medication and use of drugs, and laboratory studies equivalent to the procedures in standard clinical care).
Genotyping: For eligible patients relevant genetic variants in *CYP2D6* and *CYP2C19* will be determined. These variants explain in 90-95% of the cases with changes in CYP2D6 and CYP2C19 enzyme activity.
Based on the drug choice and based on the latest allele definition tables (KNMP kennisbank achtergrondteksten) each patient will be classified into a metabolic phenotype category (PM,IM,EM or UM).
The time period between visits 1 and 2 will be used for washout of psychiatric drugs to which insufficient response occurred and/or that interfere with study medication (also in accordance to standard clinical procedures). Genotype information and the consequent drug dosing advice will be available within five working days after visit 1.

Visit 2: Baseline (start TCA therapy). Completion of baseline assessments of secondary clinical outcome measures. During this visit TCA treatment will be started according to the instructions per treatment strategy group (genotype guided dosing vs. dosing as usual). TCAs will be initially prescribed once daily in the evening, although the dosing regimen can later on be altered according to the patient preference. The TCAs will be prescribed and delivered by either the in-hospital or outpatient pharmacy, dependent on the situation of the patient.

Visits 3 to 9 treatment phase: During seven weeks the patients receive TCA treatment based on the dosing instructions as described above (Strategies 1 and 2). Patient assessments will take place weekly for (a) TCA concentration measurements if indicated (see description) (b) the secondary clinical outcome measures as described under visit 1 (side effects and depressive symptoms). Adherence to drug treatment will be checked weekly either by review of a drug diary kept by the patient or by interviewing the patient during visits.

ECONOMIC EVALUATION (COST EFFECTIVENESS).
We aim to prospectively study cost effectiveness of the intervention during 26 weeks. This period is longer than the 7 weeks of the clinical trial, because we assume that important positive effects on patient important outcomes (quality of life and cost savings) can also be expected beyond this period.
The impact of the intervention on the quality of life of patients will be assessed both by the EuroQol 5 dimensions with 5 levels (EQ5D5L) and the SF36 at weeks 0, 2, 4, 6,13 and 26 following randomization. Primarily, utility will be derived from the EQ5D5L as recommended by the Dutch guidelines for cost-effectiveness study. However, because SF36 seems to be more valid for this particular patient population because it includes more items within the mental health domain, utility will also be derived using the SF36 system as a secondary analysis. The derived utility will be used to estimate a Quality adjusted life year (QALY) according to the trapezium rule.
The cost analysis consists of two main parts. First, at patient level, volumes of care related to MDD and TCA therapy will be measured by means of the iMTA Medical Consumption Questionnaire. This questionnaire measures all relevant health care related costs like outpatient visits at any medical specialist and hospitalizations. In addition the medication use will be derived from the electronic patient records. Loss of productivity due to illness or recovery, will be estimated based on patient reported absences from paid (or unpaid) labor measured with the Productivity Cost Questionnaire. The iMTA Medical Consumption Questionnaire and the Productivity Cost Questionnaire will be administered at the start of the study (day 0) at week 13 and at week 26.
The second part of the cost analysis consists of determining the cost prices for each volume of consumption. The standard cost prices from the 'Dutch Guidelines for Cost Analyses' and www.medicijnkosten.nl will be used. For units of care where no standard prices are available real costs prices will be determined on the basis of full cost pricing. Productivity losses will be

valued by means of the friction cost method. In the end volumes of care will be multiplied with the cost prices for each volume of care to calculate costs.
For reasons of efficiency of the trial we target in this study equally sized groups with specific genotypes. However for the cost-effectiveness analyses we would like to calculate expected values for the population of depressed patients incorporating the real prevalence of poor (PM), ultra (UM) and intermediate (IM) metabolizers opposed to normal metabolizers (EM). Therefore we will combine the prospectively gathered information on costs and utilities in this trial with literature on the prevalence of PM, UM and IM into a static decision analytic model. Ultimately one incremental cost-utility ratio's (ICUR) comparing genotype guided dosing with usual care, expressed as costs per QALY gained, will be calculated. The model based cost-effectiveness analysis will be carried out using probabilistic sensitivity analysis, by Monte Carlo simulation, to take all uncertainties surrounding the input parameters into account. The costs are included across a gamma distribution, the probabilities and utilities with beta distributions, which is according the guidelines of the ISPOR. The results of the 5000 simulations will be plotted in cost-effectiveness planes and in willingness to pay curves.

Budget Impact Analysis (BIA)
A BIA will be performed according to the ISPOR principles of Good Practice for Budget Impact Analysis. This BIA allows prediction of the financial consequences related to the adaption and implementation of the genotype guided dosing strategy in severely depressed patients, in order to determine the affordability of the intervention. Data will be used that reflect the size and characteristics of the depressed population in the Netherlands together with the results of this trial (effect sizes, resource use etc). When relevant, budget impact analyses are generated as a series of scenario analyses.

6.4 Withdrawal of individual subjects

Subjects can leave the study at any time for any reason if they wish to do so without any consequences. The investigator can decide to withdraw a subject from the study for urgent medical reasons.

6.4.1 Specific criteria for withdrawal (if applicable)

Criteria for withdrawal are:

1. when, according to the treating physician, a different biological treatment than TCA-pharmacotherapy is urgently needed (for instance electroconvulsive therapy (ECT) in case of active suicidal behaviour) .
2. Intolerable adverse reactions.
3. Severely decreasing somatic condition
4. Allergic reactions.

6.5 Replacement of individual subjects after withdrawal

Patients will not be replaced as withdrawal is accounted for in the estimation of the number of subjects necessary for screening.

6.6 Follow-up of subjects withdrawn from treatment

The data of withdrawn patients will be included in the analyses based on the intention-to-treat model. If possible we will keep withdrawn patients in the study for the part of the economic evaluations.

6.7 Premature termination of the study not applicable.

Not applicable.

7. SAFETY REPORTING

This study is not evaluating the TCAs per se, but we have decided to apply the safety regulations for AEs and SAEs that are common in clinical trials.

7.1 Temporary halt for reasons of subject safety

In accordance to section 10, subsection 4, of the WMO, the sponsor will suspend the study if there is sufficient ground that continuation of the study will jeopardise subject health or safety. The sponsor will notify the accredited METC without undue delay of a temporary halt including the reason for such an action. The study will be suspended pending a further positive decision by the accredited METC. The investigator will take care that all subjects are kept informed.

7.2 AEs, SAEs and SUSARs

7.2.1 Adverse events (AEs)

Adverse events are defined as any undesirable experience occurring to a subject during the study, whether or not considered related to [the investigational product / trial procedure/ the experimental intervention]. All adverse events reported spontaneously by the subject or observed by the investigator or his staff will be recorded.

7.2.2 Serious adverse events (SAEs)

A serious adverse event is any untoward medical occurrence or effect that

- results in death;
- is life threatening (at the time of the event);
- requires hospitalisation or prolongation of existing inpatients' hospitalisation;
- results in persistent or significant disability or incapacity;
- is a congenital anomaly or birth defect; or
- any other important medical event that did not result in any of the outcomes listed above due to medical or surgical intervention but could have been based upon appropriate judgement by the investigator.

An elective hospital admission will not be considered as a serious adverse event.

The investigator will report all SAEs to the sponsor without undue delay after obtaining knowledge of the events.

The sponsor will report the SAEs through the web portal *ToetsingOnline* to the accredited METC that approved the protocol, within 7 days of first knowledge for SAEs that result in death or are life threatening followed by a period of maximum of 8 days to complete the initial preliminary report. All other SAEs will be reported within a period of maximum 15 days after the sponsor has first knowledge of the serious adverse events.

Suspected unexpected serious adverse reactions (SUSARs): not applicable

7.3 Annual safety report

not applicable.

7.4 Follow-up of adverse events

All AEs will be followed until they have abated, or until a stable situation has been reached. Depending on the event, follow up may require additional tests or medical procedures as indicated, and/or referral to the general physician or a medical specialist. SAEs need to be reported till end of study within the Netherlands, as defined in the protocol

7.5 [Data Safety Monitoring Board (DSMB) / Safety Committee]

We do not expect that patients in this study are exposed to extra risks besides the risks of venepuncture. Instead we expect lower risk levels (see Summary section, paragraph on Burden and Risks, and see the following paragraph 11 on Structured risk analysis). A DSMB is not needed in this study.

8. STATISTICAL ANALYSIS

DATA-ANALYSIS Patient characteristics will be summarized as means or percentages depending on the type of measurement. All analyses are performed according to an intention-to-treat principle.

8.1 Primary study parameter(s)

For the measurement of differences in the primary study parameter (time needed to reach a plasma level in the therapeutic range) we will report survival probabilities according to the Kaplan-Meier method together with 95% confidence intervals (CIs). Results will be compared using two-sided log-rank test statistics. Median survival times (MST) will be presented. Patients who have not reached the study endpoint after 7 weeks will be censored to the time point of their most recent plasma level assessment.

8.2 Secondary study parameter(s)

The secondary continuous outcomes will be analysed by either student's t-test or Mann Withney U test dependent on the normal distribution of data or not. For the HAM-D scores we will compute the reduction as the baseline scores minus the scores at 7 weeks of treatment. FIBSER scores for side effects will be reported as means and standard deviations; qualitative details of the side effects will also be provided. For all statistical test we will use a significance level of 5% (two-sided). For missing values we will use the most recent HAM-D score that is available before the end of the medication study (7 weeks). For the FIBSER we will use the highest level of side effects measured during the medication study.

In additional analyses we will compare the primary and secondary outcome measures of the deviant metabolizer groups with those of patients with the normal metabolization phenotype.

Multiple regression analysis will be used to study the predictive effects of the early TCA serum/plasma level and other clinical (psychomotor retardation) and biological factors on the primary and secondary outcome measures (see research questions).

8.3 Other study parameters

For the economic analysis see also the description above (section 6.3). Here we will use a mixed repeated measures design. Missing values will be interpolated between the available assessments.

8.4 Interim analysis (if applicable)

Not applicable

9. ETHICAL CONSIDERATIONS

9.1 Regulation statement

The study will be conducted according to the principles of the Declaration of Helsinki (version October 2008) and in accordance with the Medical Research Involving Human Subjects Act (WMO).

9.2 Recruitment and consent

Participants will be guaranteed privacy, anonymity, and confidentiality, and will be told explicitly that they are allowed to withdraw from the study at any time. Furthermore, they will be informed that the researchers have the right to exclude them from participation at any time. The information will be given to participants in the form of a written patient information brochure containing also the informed consent form. At least 24 hours after receiving this information, potential participants will be contacted again by telephone. This will give the potential participant time to reflect on the study and the implications of consent. Any remaining questions of the potential participant will be answered and the researcher will perform a first exclusion screening based on the pre-screening questionnaire that is part of the information brochure. If there is no reason to exclude the potential participant based on these questions, the appointment will be made. Before inclusion, all participants are required to sign the informed consent form.

9.3 Objection by minors or incapacitated subjects (if applicable)

Not applicable

9.4 Benefits and risks assessment, group relatedness

Not applicable

9.5 Compensation for injury

The sponsor/investigator has a liability insurance which is in accordance with article 7 of the WMO.

The sponsor (also) has an insurance which is in accordance with the legal requirements in the Netherlands ((Article 7 WMO and the Measure regarding Compulsory Insurance for

Clinical Research in Humans of 23th June 2015). This insurance provides cover for damage to research subjects through injury or death caused by the study.

For details of these insurances we refer to the appendices G2 and G1 respectively.

9.6 Incentives (if applicable)

As our study is executed in the context of common clinical practice, patients will not be offered specific incentives.

ADMINISTRATIVE ASPECTS, MONITORING AND PUBLICATION

9.7 Handling and storage of data and documents

The investigator will ensure that the subject's anonymity will be maintained. On all documents subjects will be identified only by an identification code – not by their names or hospital/clinical number. The investigator will keep a separate Subject Identification Code List, which matches identifying codes with the subject's names. Documents will be maintained by the investigator in strict confidence. Subject anonymity has to maintain upon all archiving steps. After cessation of the whole study (finalized and signed research report) the investigator will store the investigator file and copies of the CRFs to fulfill his responsibility to maintain adequate human study records for at least 2 years after the final publication or longer if required by the applicable legislation. Handling of personal data will comply with the Dutch Personal Data Protection Act. Subjects will be given a personal identification number, based on a random number generator. The executive investigator only will have the key to the code and will be able to track back data to personal subjects. Personal data will be kept separately from the experimental data acquired.

All biological materials will be coded on the patient's identification number and stored at the department of Human genetics Radboudumc according to the department's storage procedures. Only Dr. M.J.H. Coenen and Dr. J. Janzing will have access to the key of the code. All materials will be stored for 25 years. After this period and in case of withdrawal of the patients (on their request) the materials will be destroyed. Aim of storage is to be able to perform additional analyses. If analyses are suggested which are beyond the scope of the current informed consent we will request for additional permission at the CMO (amendment to the current protocol) and we will approach the patients for a separate written consent. **The CMO will also be consulted before genetic analyses will performed which include a risk of incidental findings**

Biomaterials will not be released unless there is a separate permission by the CMO (permission after request).

9.8 Monitoring and Quality Assurance

Before the start of the study a monitoring plan will be developed under the supervision of the clinical research centre Nijmegen.

Monitoring will be performed in this study by a GCP certified monitor. Prior to first screening an initiation visit will be performed with the site (CRCN) and after the trial a close out visit will be performed with each study site.

The monitoring will consists of:
- check essential documents at the site
- check eligibility of subjects prior to study start
- monitoring for completeness and correctness of the source documents
- monitoring of the data in the CRCN workbook, and transfer of data from source documents to the eCRF (database management system CASTOR).

Checklists will be used containing the most important instructions.

9.9 Annual progress report

The sponsor/investigator will submit a summary of the progress of the trial to the accredited METC once a year. Information will be provided on the date of inclusion of the first subject, numbers of subjects included and numbers of subjects that have completed the trial, serious adverse events/ serious adverse reactions, other problems, and amendments.

9.10 Temporary halt and (prematurely) end of study report

The investigator/sponsor will notify the accredited METC of the end of the study within a period of 8 weeks. The end of the study is defined as the last patient's last visit.

The sponsor will notify the METC immediately of a temporary halt of the study, including the reason of such an action.

In case the study is ended prematurely, the sponsor will notify the accredited METC within 15 days, including the reasons for the premature termination.

Within one year after the end of the study, the investigator/sponsor will submit a final study report with the results of the study, including any publications/abstracts of the study, to the accredited METC.

10. Public disclosure and publication policy

The study will be registered at **https://clinicaltrials.gov**
The results will be published in international scientific peer reviewed journals.
The public disclosure and publication of the research data will be in agreement with the guidelines of ZonMW. Below we present our strategies in more detail.

To achieve implementation the results of our study must quickly find their way to clinicians, clinical genetic physicians and pharmacists and patients. In a collaborative, interdisciplinary effort we aim to submit our results to national and international peer reviewed journals and present them on national and international congresses, public and patient symposia and workshops.

We expect that our study results will be used to inform treatment recommendations as expressed in future clinical guidelines. This will be facilitated by the national and international positions held by our team members and advisors (among others): dr. R. van Westrhenen chairs the Dutch Psychiatric Association (NVvP) workgroup preparing the guidelines for pharmacogenetics based dosing and Therapeutic Drug Monitoring. Professor Schene chairs the Dutch Program for Quality Networks, coordinating the development of all National Guidelines in Mental Health. Professor Spijker chairs the “Zorgstandaard Depressie” (National Depression Guideline) and is editor of the Dutch and Flemish Journal of Psychiatry (Tijdschrift voor Psychiatrie). Professor van Schaik and dr Swen are members of the Pharmacogenetics workgroup of the KNMP. Drs. Coenen, Swen and Prof. van Schaik are board members of the Dutch Clinical Pharmacogenetics Network. Prof. van Schaik is the President of the European Society for Pharmacogenomics & Personalised Therapy (ESPT). Prof. van Gelder is the chairman of the Dutch Society for Clinical Pharmacology and the President-Elect of the International Society for Therapeutic Drug Monitoring and Clinical Toxicology.

All investigators have extensive experience in publication in national and international peer reviewed journals and take actively part in the local, regional and national education programmes for psychiatrists, clinical genetics physicians, pharmacists, general medical doctors and other health professionals.

11. STRUCTURED RISK ANALYSIS

Potential issues of concern. The only extra risk participants are exposed to is the risk associated with the extra venepunctures. The aim of the study is to decrease the risk associated with CYP450 unadjusted prescription of TCA's. In the intervention group we therefore expect lower chances for side effects and a reduced time to therapeutic response compared to treatment as usual. The weekly monitoring for side effects and depressive symptoms in all participating patients give a low burden to the patients but contribute to extra safety compared to regular care.
Because the only risk is determined by the venepunctures the items below are not applicable.

The following section is not applicable.

a. Level of knowledge about mechanism of action

b. Previous exposure of human beings with the test product(s) and/or products with a similar biological mechanism

c. Can the primary or secondary mechanism be induced in animals and/or in *ex-vivo* human cell material?

d. Selectivity of the mechanism to target tissue in animals and/or human beings

e. Analysis of potential effect

f. Pharmacokinetic considerations

g. Study population

h. Interaction with other products

<u>i. Predictability of effect</u>

j. Can effects be managed?

11.1 Synthesis

As already explained the only risk consists of the extra vene punctures.

12. REFERENCES

American Psychiatric Association (APA). Practice guideline for the treatment of Patients With Major Depressive Disorder Third Edition 2010. http://psychiatryonline.org/pb/assets/raw/sitewide/practice_guidelines/guidelines/mdd.pdf

Birkenhäger and Moleman. Psyfar april 2007: 13-16.

Chekroud AM, Krystal JH. BMJ. 2015 May 14;350:h2502.

Coenen MJ et al. Gastroenterology. 2015 Oct;149(4):907-17.e7.

Farmacotherapeutisch Kompas. http://www.farmacotherapeutischkompas.nl/

Grove DS. J Biomol Tech. 1999 Mar;10(1):11-6.

Hamilton M. J Neurol Neurosurg Psychiatry. 1960 Feb;23:56-62.

Hicks JK et al. Clin Pharmacol Ther. 2016 Dec 20;. doi: 10.1002/cpt.597 [Epub ahead of print]

Hiemke C et al. Pharmacopsychiatry. 2011 Sep;44(6):195-235. Review

Ingelman-Sundberg M. Pharmacogenomics J. 2005;5(1):6-13.

Johnson JA et al. Clin Pharmacol Ther. 2013 May;93(5):384-5

Kessler RC, Bromet EJ. Annu Rev Public Health. 2013;34:119-38.

L.M.Lamers, P.F.M.Stalmeier, J.McDonnell, P.F.M.Krabbe en J.J.van BusschbachKwaliteit van leven meten in economische evaluaties: het Nederlands EQ-5D-tarief; Ned Tijdschr Geneeskd 2005;149:1574-8

Multidisciplinaire richtlijn depressie (MDR) 3e revisie 2013. Via: http://www.ggzrichtlijnen.nl/

Onderzoeksagenda GGz (februari 2016) De juiste behandeling op het juiste moment.

Ruhé HG et al. J Affect Disord. 2012 Mar;137(1-3):35-45. 2.

Rijksuniversiteit Groningen. Nederlandse vertaling van de RAND 36-item health survey 1.0 (RAND, 1992). 1993.

Rush AJ et al. Psychiatry Research 1986; 18(1): 65-87

Rush AJ et al. Am J Psychiatry. 2006 Nov;163(11):1905-17.

Schenk PW et al. Mol Psychiatry. 2008 Jun;13(6):597-605

Schenk PW et al. Pharmacogenomics J. 2010 Jun;10(3):219-25.

Swen JJ et al. Clin Pharmacol Ther. 2011 May;89(5):662-73.

Swen JJ et al. PLoS Med. 2007 Aug;4(8):e209.

Uher-R et al. Br J Psychiatry. 2009 Sep;195(3):202-10.

Widlöcher D, Ghozlan A. The measurement of retardation in depression. In: Hindmarch I, Stonier PD, editors.Human Psychopharmacology: Measures and Methods. Wiley, New York, 1989.

Wisniewski SR et al. J Psychiatr Pract. 2006 Mar;12(2):71-9.

APPENDIX A. VARIANTS FOR GENOTYPING

Genotype	Variation	RS number	Gene location	Assay ID
CYP2C19 *2	681 G>A	RS4244285	Exon 5	C__25986767_70
CYP2C19 *3	636 G>A	RS4986893	Exon 4	C__27861809_10
CYP2C19 *17	-806 C>T	RS12248560	5'-UTR	C____469857_10
CYP2D6*1 (WT)				
CYP2D6*2	2850C>T,<br>4180G>C			
CYP2D6 *3	2549 delA	RS35742686	Exon 5	C__32407232_50
CYP2D6 *4	1846 G>A	RS3892097	Exon 4	C__27102431_B0
CYP2D6 *5	Gene deletion		All exons	Hs00010001_cn
CYP2D6 *6	1707 delT	RS5030655	Exon 3	C__32407243_20
CYP2D6*7	2935A>C	rs5030867		
CYP2D6*8	1758G>T	rs5030865	Intron	
CYP2D6*9	2615-2617delAAG	rs5030656		
CYP2D6*10	100C>T	rs1065852		
CYP2D6*15	137insT			
CYP2D6*17	1023C>T	rs28371706		
CYP2D6*29	1659G>A;<br>1661G>C;<br>2850C>T;<br>3183G>A;<br>4180G>C	rs61736512(T)<br>rs1058164(C)<br>rs16947(A)<br>rs59421388(T)<br>rs1135840(C)		
CYP2D6*35				
_CYP2D6_*41	2988 G>A	RS28371725	Intron 6	C__34816116_20
CYP2D6 xn	Gene duplication		All exons	Hs00010001_cn

APPENDIX B.

De bepaling van de metabolisatiefenotypen op basis van de uitslagen van het genetisch onderzoek zal gebeuren volgens de richtlijnen

Algemene achtergrondtekst Farmacogenetica - CYP2D6

Algemene achtergrondtekst Farmacogenetica - CYP2C19

Welke zijn te vinden op www.kennisbank.knmp.nl

APPENDIX C. Pharmacogenetics based dose advice (KNMP see www.kennisbank.knmp.nl).

Gene	**CYP2D6**				**CYP2C19**			
Metaboliser status	UM	EM	IM	PM	UM	EM	IM	PM
Clomipramine	increase dose to 150% of normal	normal dose	decrease dose to 70% of normal	decrease dose to 50% of normal	-	-	-	-
Imipramine	increase dose to 170% of normal	normal dose	decrease dose to 70% of normal	decrease dose to 30% of normal	normal dose	normal dose	normal dose	decrease dose to 70% of normal
Nortriptyline	increase dose to 160% of normal	normal dose	decrease dose to 60% of normal	decrease dose to 40% of normal	-	-	-	-

In this study we primarily follow KNMP guidelines, as these guidelines do not contain dosing guidance for patients with both *CYP2D6* and *CYP2C19* deviant metabolizer status we follow table 4 from Hicks et al. (2016). This table of Hicks et al. (2016) assumes additive effects in patients with double deviant metabolizer status. Clinical pharmacogenetics implementation consortium guideline (CPIC) for CYP2D6 and CYP2C19 genotypes and dosing of tricyclic antidepressants: Clin Pharmacol Ther. 2016 Dec 20. doi: 10.1002/cpt.597.
