# Supplementary material for: Effectiveness of Genotype-Specific Tricyclic Antidepressant Dosing in Patients With Major Depressive Disorder: A Randomized Clinical Trial
Source: JAMA Netw Open. 2023 May 8;6(5):e2312443. doi: 10.1001/jamanetworkopen.2023.12443 (PMC10167565; doi:10.1001/jamanetworkopen.2023.12443)
Supplement: Supplement 2. — eTable 1. Number of Patients per Inclusion Center, Presented as Numbers and Percentages eTable 2. Nortriptyline and Clomipramine Dosing Advice per CYP2D6 Metabolizer Phenotype According to DPWG Guidelines eTable 3. Imipramine Dosing Advice per CYP2D6 and CYP2C19 Metabolizer Phenotype According to DPWG Guidelines eTable 4. Standard Dosing Advice for Nortriptyline, Clomipramine, and Imipramine According to the Dutch Dosing Guideline (https://www.farmacotherapeutischkompas.nl/) eFigure 1. Comparison of TCA Plasma Concentrations Corresponding to PIT and TAU Dosing Advice per CYP2D6 Phenotype for (A) Nortriptyline, (B) Clomipramine, and (C) Imipramine, Presented as Tukey Plots and as Means With SDs eFigure 2. Comparison of TCA Plasma Concentrations in PIT and TAU Groups per CYP2C19 Phenotype for (A) Nortriptyline, (B) Clomipramine, and (C) Imipramine, Presented as Tukey Plots and as Means With SDs eFigure 3. Depression Severity (HAMD-17 Score) and Severity of Adverse Effects (FIBSER Item 2) for (A) Nortriptyline (n = 67) and (B) Clomipramine (n = 38), Presented as Means With SEs eFigure 4. Comparison Between PIT (n = 56) and TAU (n = 55) Regarding (A) Frequency (FIBSER Item 1) and (B) Burden of Adverse Effects (FIBSER Item 3), Presented as Means With SEs eTable 5. Baseline Characteristics of the Reference Group (n = 46) Compared With the PIT (n = 56) and TAU (n = 55) Groups [file jamanetwopen-e2312443-s002.pdf]

Supplementary Online Content

Vos CF, ter Hark SE, Schellekens AFA, et al. Effectiveness of genotype-specific tricyclic antidepressant dosing in patients with major depressive disorder: a randomized clinical trial. *JAMA Netw Open*. 2023;6(5):e2312443. doi:10.1001/jamanetworkopen.2023.12443

eTable 1. Number of Patients per Inclusion Center, Presented as Numbers and Percentages

eTable 2. Nortriptyline and Clomipramine Dosing Advice per CYP2D6 Metabolizer Phenotype According to DPWG Guidelines

eTable 3. Imipramine Dosing Advice per CYP2D6 and CYP2C19 Metabolizer Phenotype According to DPWG Guidelines

eTable 4. Standard Dosing Advice for Nortriptyline, Clomipramine, and Imipramine According to the Dutch Dosing Guideline (https://www.farmacotherapeutischkompas.nl/)

eFigure 1. Comparison of TCA Plasma Concentrations Corresponding to PIT and TAU Dosing Advice per CYP2D6 Phenotype for (A) Nortriptyline, (B) Clomipramine, and (C) Imipramine, Presented as Tukey Plots and as Means With SDs

eFigure 2. Comparison of TCA Plasma Concentrations in PIT and TAU Groups per CYP2C19 Phenotype for (A) Nortriptyline, (B) Clomipramine, and (C) Imipramine, Presented as Tukey Plots and as Means With SDs

eFigure 3. Depression Severity (HAMD-17 Score) and Severity of Adverse Effects (FIBSER Item 2) for (A) Nortriptyline (n = 67) and (B) Clomipramine (n = 38), Presented as Means With SEs

eFigure 4. Comparison Between PIT (n = 56) and TAU (n = 55) Regarding (A) Frequency (FIBSER Item 1) and (B) Burden of Adverse Effects (FIBSER Item 3), Presented as Means With SEs

eTable 5. Baseline Characteristics of the Reference Group (n = 46) Compared With the PIT (n = 56) and TAU (n = 55) Groups

This supplemental material has been provided by the authors to give readers additional information about their work.

© 2023 Vos CF et al. *JAMA Network Open*.

eTable 1. Number of Patients per Inclusion Center, Presented as Numbers and Percentages

Inclusion center	Total	PIT[a]	TAU[b]
Radboudumc Nijmegen	137 (80.1%)	49 (87.5%)	42 (76.4%)
Pro Persona Nijmegen, Tiel and Ede	31 (18.1%)	6 (10.7%)	11 (20.0%)
Vincent van Gogh Venlo	2 (1.2%)	1 (1.8%)	1 (1.8%)
Catharina Hospital Eindhoven	1 (0.6%)	0 (0.0%)	1 (1.8%)
Total	171	56	55

[a]PIT = Pharmacogenetics-Informed Treatment, [b]TAU = Treatment As Usual

© 2023 Vos CF et al. *JAMA Network Open*.

eTable 2. Nortriptyline and Clomipramine Dosing Advice per CYP2D6 Metabolizer Phenotype According to DPWG Guidelines

Dose adjustments are presented in milligrams/day (mg/day) and as percentage (%) of the standard dose.

CYP2D6 metabolizer phenotype	nortriptyline	clomipramine
CYP2D6 UM	200 mg/day (160%)	187.5 mg/day (150%)
CYP2D6 NM	125 mg/day (100%)	125 mg/day (100%)
CYP2D6 IM	75 mg/day (60%)	85 mg/day (70%)
CYP2D6 PM	50 mg/day (40%)	60 mg/day (50%)

PM = Poor metabolizer, IM = Intermediate metabolizer, NM = Normal metabolizer, UM = Ultrarapid metabolizer.

© 2023 Vos CF et al. *JAMA Network Open*.

eTable 3. Imipramine Dosing Advice per CYP2D6 and CYP2C19 Metabolizer Phenotype According to DPWG Guidelines

Dose adjustments are presented in milligrams/day (mg/day) and as percentage (%) of the standard dose.

	CYP2C19 UM	CYP2C19 NM	CYP2C19 IM	CYP2C19 PM
CYP2D6 UM	175 mg/day (170%)	175 mg/day (170%)	175 mg/day (170%)	Start nortriptyline
CYP2D6 NM	175 mg/day (100%)	175 mg/day (100%)	175 mg/day (100%)	125 mg/day (70%)
CYP2D6 IM	125 mg/day (70%)	125 mg/day (70%)	125 mg/day (70%)	Start nortriptyline
CYP2D6 PM	50 mg/day (30%)	50 mg/day (30%)	50 mg/day (30%)	Start nortriptyline

PM = Poor metabolizer, IM = Intermediate metabolizer, NM = Normal metabolizer, UM = Ultrarapid metabolizer.

© 2023 Vos CF et al. *JAMA Network Open*.

eTable 4. Standard Dosing Advice for Nortriptyline, Clomipramine, and Imipramine According to the Dutch Dosing Guideline (https://www.farmacotherapeutischkompas.nl/)

nortriptyline	clomipramine	imipramine
Start with 25 mg 2-3 times a day and build up to 100-150 mg/day	Start with 25 mg 2-3 times a day and build up to 100-150 mg/day	Start with 25 mg 1-3 times a day and build up to 150-200 mg/day

© 2023 Vos CF et al. *JAMA Network Open*.

eFigure 1. Comparison of TCA Plasma Concentrations Corresponding to PIT and TAU Dosing Advice per CYP2D6 Phenotype for (A) Nortriptyline, (B) Clomipramine, and (C) Imipramine, Presented as Tukey Plots and as Means With SDs

Dotted lines mark the therapeutic window.

A. Nortriptyline

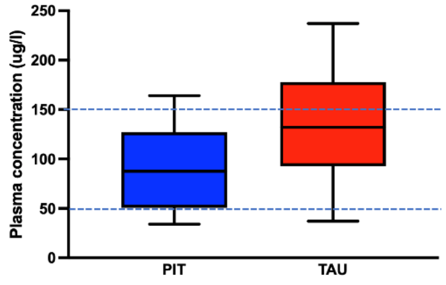

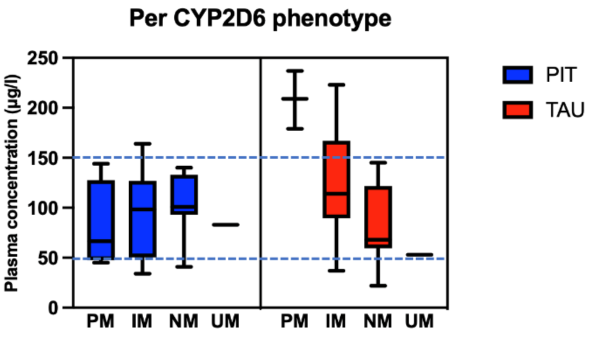

Phenotype	PIT[a]	*n*	TAU[a]	*n*	Difference[b]
CYP2D6 PM	80.5 (44.5)	4	208.3 (29.0)	3	-127.8 (-199.6 to -56.1)
CYP2D6 IM	94.5 (43.4)	18	122.8 (50.9)	17	-28.3 (-61.0 to 4.4)
CYP2D6 NM	105.2 (29.0)	11	83.5 (38.3)	10	21.7 (-10.1 to 53.5)
CYP2D6 UM	83.0 (0.0)	1	53.0(0.0)	1	30.0

[a]Plasma concentration Nortriptyline (ug/l), mean (*SD*)
[b]Mean (95% confidence interval)
PM = Poor metabolizer, IM = Intermediate metabolizer, NM = Normal metabolizer, UM = Ultrarapid metabolizer, NA = Not Available

© 2023 Vos CF et al. *JAMA Network Open*.

B. Clomipramine

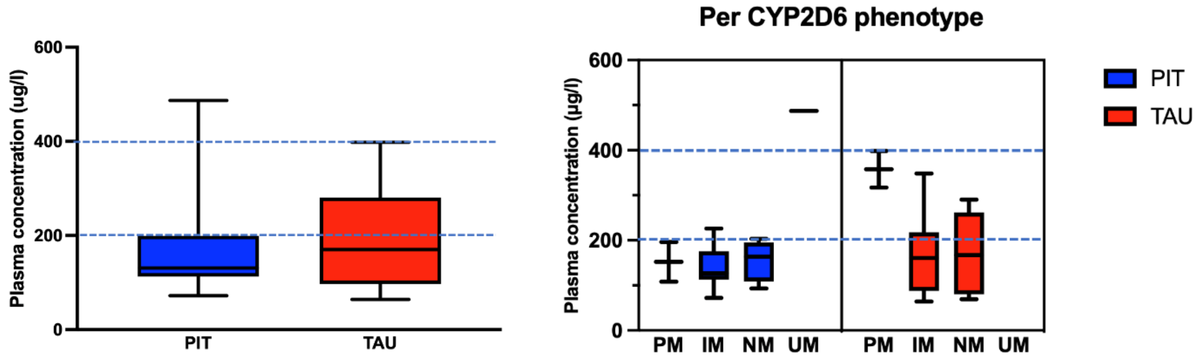

Phenotype	PIT[a]	*n*	TAU[a]	*n*	Difference[b]
CYP2D6 PM	152.0 (62.2)	2	375.5 (57.3)	2	-223.5 (-464.5 to 53.5)
CYP2D6 IM	140.5 (49.8)	10	172.0 (91.6)	9	-31.5 (-111.4 to 48.4)
CYP2D6 NM	155.8 (46.3)	4	171.5 (88.0)	6	-15.7 (-114.8 to 83.3)
CYP2D6 UM	487 (0.0)	1	NA	0	NA

[a] Sum plasma concentration Clomipramine plus Desmethylclomipramine (ug/l), mean (SD)
[b]Mean (95% confidence interval)
PM = Poor metabolizer, IM = Intermediate metabolizer, NM = Normal metabolizer, UM = Ultrarapid metabolizer, NA = Not Available

© 2023 Vos CF et al. *JAMA Network Open*.

C. Imipramine

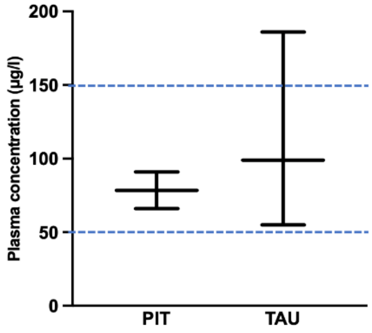

Phenotype	PIT[a]	*n*	TAU[a]	*n*	Difference[b]
CYP2D6 PM	NA	0	NA	0	NA
CYP2D6 IM	66.0 (0.0)	1	186.0 (0.0)	1	-120.0
CYP2D6 NM	91.0 (0.0)	1	77.0 (31.1)	2	14.0
CYP2D6 UM	NA	0	NA	0	NA

[a]Sum plasma concentration Imipramine plus Desipramine (ug/l), mean (SD)
[b]Mean (95% confidence interval)
PM = Poor metabolizer, IM = Intermediate metabolizer, NM = Normal metabolizer, UM = Ultrarapid metabolizer, NA = Not Available

Reference group

In the reference group (*n*=46), consisting of non-randomized patients with a CYP2D6 normal metabolizer phenotype receiving standard treatment, in 34 patients a plasma concentration was measured: 19 patients treated with nortriptyline, mean 80.7 (*SD* 32.7) ug/l, and 15 patients treated with clomipramine, mean 170.3 (*SD* 91.9).

© 2023 Vos CF et al. *JAMA Network Open*.

eFigure 2. Comparison of TCA Plasma Concentrations in PIT and TAU Groups per CYP2C19 Phenotype for (A) Nortriptyline, (B) Clomipramine, and (C) Imipramine, Presented as Tukey Plots and as Means With SDs

Dotted lines mark the therapeutic window.

A. Nortriptyline

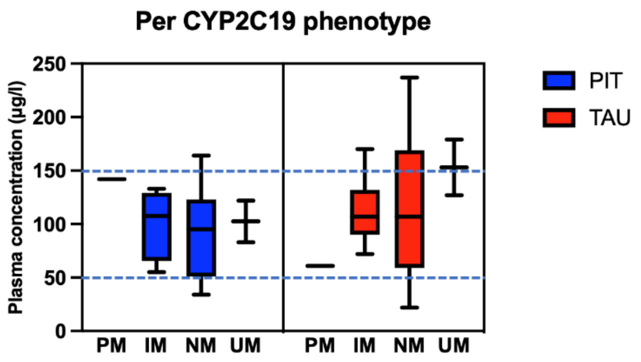

Phenotype	PIT[a]	*n*	TAU[a]	*n*	Difference[b]
CYP2C19 PM	142.0 (0.0)	1	61.0 (0.0)	1	81.0
CYP2C19 IM	100.8 (33.9)	4	113.0 (31.4)	7	-12.2 (-63.0 to 38.5)
CYP2C19 NM	93.1 (40.1)	27	116.3 (65.0)	21	-23.2 (-56.1 to 9.7)
CYP2C19 UM	102.5 (27.6)	2	153.0 (36.8)	2	-50.5 (-201.4 to 100.4)

[a]Plasma concentration Nortriptyline (ug/l), mean (*SD*)
[b]Mean (95% confidence interval)
PM = Poor metabolizer, IM = Intermediate metabolizer, NM = Normal metabolizer, UM = Ultrarapid metabolizer, NA = Not Available

© 2023 Vos CF et al. *JAMA Network Open*.

B. Clomipramine

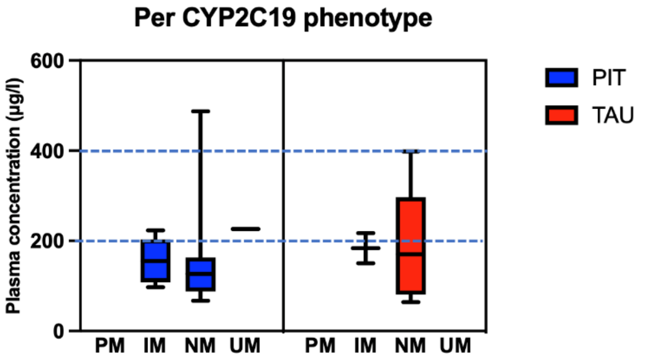

Phenotype	PIT[a]	*n*	TAU[a]	*n*	Difference[b]
CYP2C19 PM	91.0 (0.0)	1	NA	0	NA
CYP2C19 IM	158.1 (50.0)	7	183.5 (47.4)	2	-25.4 (-221.0 to 170.2)
CYP2C19 NM	165.1 (124.5)	9	196.6 (110.4)	14	-31.5 (-139.6 to 76.6)
CYP2C19 UM	226.0 (0.0)	1	NA	0	NA

[a]Sum plasma concentration Clomipramine plus Desmethylclomipramine (ug/l), mean (SD)
[b]Mean (95% confidence interval)
PM = Poor metabolizer, IM = Intermediate metabolizer, NM = Normal metabolizer, UM = Ultrarapid metabolizer, NA = Not Available

© 2023 Vos CF et al. *JAMA Network Open*.

C. Imipramine

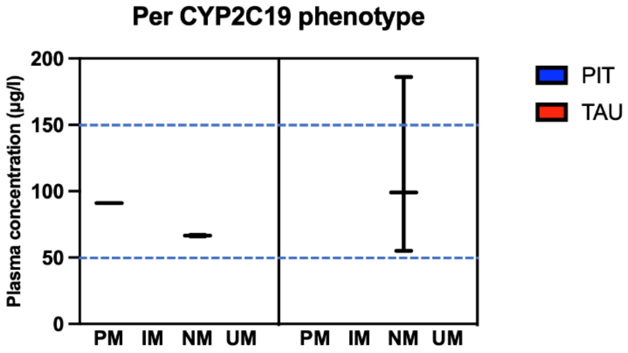

Phenotype	PIT[a]	*n*	TAU[a]	*n*	Difference[b]
CYP2C19 PM	91.0 (0.0)	1	NA	0	NA
CYP2C19 IM	NA	0	NA	0	NA
CYP2C19 NM	66.0 (0.0)	1	113.3 (66.7)	3	-47.3
CYP2C19 UM	NA	0	NA	0	NA

[a]Sum plasma concentration Imipramine plus Desipramine (ug/l), mean (SD)
[b]Mean (95% confidence interval)
PM = Poor metabolizer, IM = Intermediate metabolizer, NM = Normal metabolizer, UM = Ultrarapid metabolizer, NA = Not Available

© 2023 Vos CF et al. *JAMA Network Open*.

eFigure 3. Depression Severity (HAMD-17 Score) and Severity of Adverse Effects (FIBSER Item 2) for (A) Nortriptyline (n = 67) and (B) Clomipramine (n = 38), Presented as Means With SEs

A.   Nortriptyline

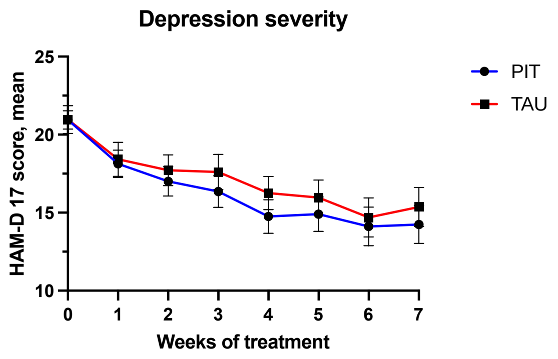

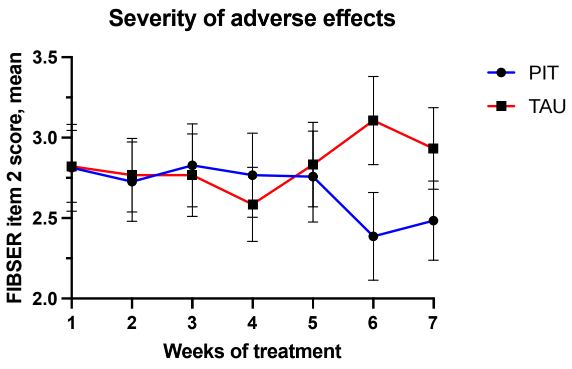

Number of patients

Week	*0*	*1*	*2*	*3*	*4*	*5*	*6*	*7*
PIT	31	32	33	34	33	31	33	33
TAU	32	31	32	30	32	31	29	30

Number of patients

Week	*1*	*2*	*3*	*4*	*5*	*6*	*7*
PIT	32	33	29	30	29	31	33
TAU	28	30	30	29	30	28	30

© 2023 Vos CF et al. *JAMA Network Open*.

B. Clomipramine

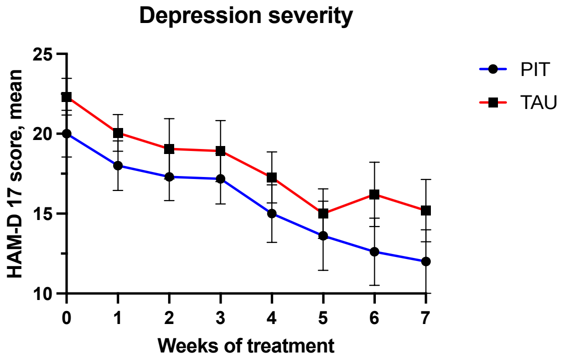

Number of patients								
Week	*0*	*1*	*2*	*3*	*4*	*5*	*6*	*7*
PIT	18	18	16	17	15	13	13	14
TAU	19	18	16	13	15	14	14	15

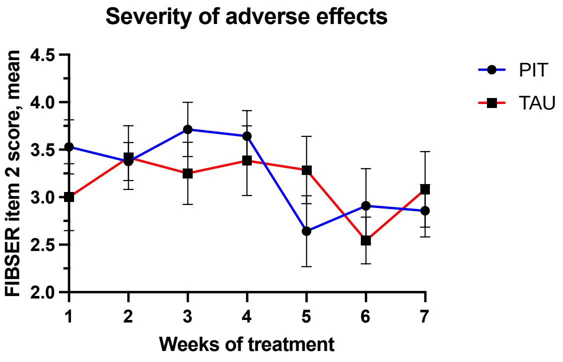

Number of patients							
Week	*1*	*2*	*3*	*4*	*5*	*6*	*7*
PIT	17	16	14	14	14	11	14
TAU	16	12	12	13	14	11	12

© 2023 Vos CF et al. *JAMA Network Open*.

eFigure 4. Comparison Between PIT (n = 56) and TAU (n = 55) Regarding (A) Frequency (FIBSER Item 1) and (B) Burden of Adverse Effects (FIBSER Item 3), Presented as Means With SEs

A

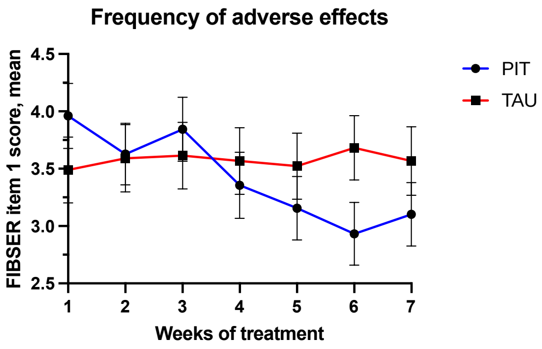

Number of patients							
Week	*1*	*2*	*3*	*4*	*5*	*6*	*7*
PIT	51	51	45	45	45	44	49
TAU	47	44	44	44	46	41	44

B

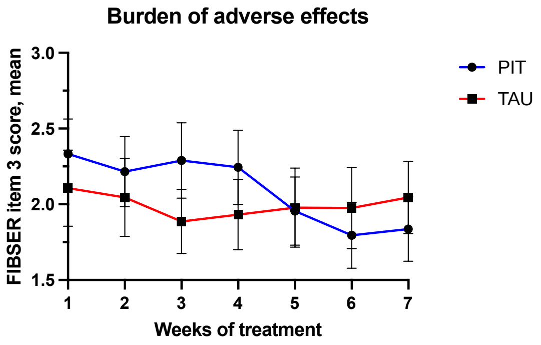

Number of patients							
Week	*1*	*2*	*3*	*4*	*5*	*6*	*7*
PIT	51	51	45	45	45	44	49
TAU	47	44	44	44	46	41	44

© 2023 Vos CF et al. *JAMA Network Open*.

eTable 5. Baseline Characteristics of the Reference Group (n = 46) Compared With the PIT (n = 56) and TAU (n = 55) Groups

	Reference group (*n*=46)	PIT (*n*=56)	TAU (*n*=55)
Gender, *n* (%)			
Female	28 (61)	36 (64)	33 (60)
Male	18 (39)	20 (36)	22 (40)
Age (years), mean (*SD*)	44.2 (14.4)	40.8 (14.1)	42.7 (12.6)
HAM-D-17 score, mean (*SD*)	22.4 (3.6)	20.8 (4.7)	21.3 (4.9)
Duration[¶], *n* (%)			
0-1 years	18 (39)	23 (41)	21 (38)
1-2 years	10 (22)	11 (20)	11 (20)
>2 years	18 (39)	22 (39)	23 (42)
Depressive episodes, *n* (%)			
First	11 (24)	17 (30)	16 (29)
Recurrent	35 (76)	39 (70)	39 (71)
CYP2D6 phenotype, *n* (%)			
PM	0 (0)	6 (11)	6 (11)
IM	0 (0)	30 (53)	27 (49)
NM	46 (100)	18 (32)	21 (38)
UM	0 (0)	2 (4)	1 (2)
CYP2C19 phenotype, *n* (%)			
PM	3 (7)	2 (4)	1 (2)
IM	6 (13)	12 (21)	10 (18)
NM	33 (72)	39 (70)	41 (75)
UM	4 (8)	3 (5)	3 (5)
TCA, *n* (%)			
Nortriptyline	27 (59)	34 (61)	33 (60)
Clomipramine	19 (41)	19 (34)	19 (35)
Imipramine	0 (0)	3 (5)	3 (5)

[¶] Duration of the current depressive episode. PM = Poor metabolizer, IM = Intermediate metabolizer, NM = Normal metabolizer, UM = Ultrarapid metabolizer.

© 2023 Vos CF et al. *JAMA Network Open*.
