# Supplementary material for: Effectiveness of Genotype-Specific Tricyclic Antidepressant Dosing in Patients With Major Depressive Disorder: A Randomized Clinical Trial
Source: JAMA Netw Open. 2023 May 8;6(5):e2312443. doi: 10.1001/jamanetworkopen.2023.12443 (PMC10167565; doi:10.1001/jamanetworkopen.2023.12443)
Supplement: Supplement 3. — Data Sharing Statement [file jamanetwopen-e2312443-s003.pdf]

Data Sharing Statement

Vos. Effectiveness of Genotype-Specific Tricyclic Antidepressant Dosing in Patients with Major Depressive Disorder. *JAMA Netw Open*. Published May 08, 2023.
doi:10.1001/jamanetworkopen.2023.12443

Data
Data available: No

Additional Information
Explanation for why data not available: Data will be made available based on reasonable request
